# Essential Oils Derived from Traditional Chinese Medicinal Herbs as Natural Antimicrobial Candidates for Foodborne Pathogen Control: Antibacterial Activity, Biofilm Removal, and Volatile Composition

**DOI:** 10.3390/foods15152675

**Published:** 2026-07-29

**Authors:** Wenbin Xiao, Xiaotong Liu, Xinjia Tan, Lihua Hu, Yunhua Xiao, Hua Yang, Huawu Zhu

**Affiliations:** 1 College of Bioscience and Biotechnology, Hunan Agricultural University, Changsha 410128, China; 2 Yuelushan Laboratory, Changsha 410128, China; 3 College of Agronomy, Hunan Agricultural University, Changsha 410128, China

**Keywords:** traditional Chinese medicinal herbs, essential oils, natural antimicrobial, foodborne pathogens, biofilm removal, volatile composition, *Lonicera japonica*

## Abstract

Foodborne pathogens and their biofilms threaten microbial safety in food systems, highlighting the need for antimicrobial candidates. This study compared nine essential oils derived from traditional Chinese medicinal herbs (TCM herb essential oils) for antibacterial activity, mature biofilm removal, volatile composition, and candidate compounds against *Staphylococcus aureus*, *Listeria monocytogenes*, *Escherichia coli*, and *Salmonella paratyphi* B using DIZ, MIC, crystal violet staining, GC–MS, growth curves, and SEM. Among the tested oils, *Lonicera japonica*, *Sinomenium acutum*, and *Scutellaria baicalensis* essential oils showed relatively broad antibacterial activity. *Lonicera japonica* essential oil (LJEO) displayed the most notable overall performance, producing DIZ values of 38.29, 25.29, and 24.75 mm against *S. aureus*, *L. monocytogenes*, and *S. paratyphi* B, respectively, and low MIC against *S. aureus* and *L. monocytogenes* at 0.15625% and 0.3125%. LJEO removed mature biofilms of *L. monocytogenes*, *E. coli*, and *S. paratyphi* B by 60.58%, 50.80%, and 47.82%, respectively, but showed limited activity against *S. aureus* biofilms. GC–MS identified 156 volatile compounds, with LJEO characterized by abundant linalool, p-cymene, borneol, cineoles, and limonene. Growth curves and SEM indicated that LJEO inhibited bacterial proliferation and damaged cell surface structures. These findings suggest that TCM herb essential oils, especially LJEO, may serve as natural antimicrobial candidates for foodborne pathogen control and food preservation.

## 1. Introduction

Microbial contamination is one of the major factors affecting food safety, food quality, and public health. Food products can be contaminated by microorganisms during raw material harvesting, processing, transportation, storage, and distribution. Among them, foodborne pathogens such as *Staphylococcus aureus*, *Listeria monocytogenes*, *Escherichia coli*, and *Salmonella* spp. are among the most concerning microbial hazards in food safety research. *S. aureus* can produce heat-stable enterotoxins and cause acute food poisoning, and its pathogenicity is closely associated with extracellular virulence factors such as α hemolysin, staphylococcal enterotoxins, toxic shock syndrome toxin 1, and other related factors [1]. *L. monocytogenes* can survive under refrigeration, high-salt, and mildly acidic conditions, and is frequently associated with contamination of refrigerated ready-to-eat foods [2,3]. *E. coli* and *Salmonella* spp. are also important pathogens involved in gastrointestinal infections and foodborne disease outbreaks [4,5]. Recent estimates released by the World Health Organization further indicate that unsafe food causes a substantial global burden of illness and death each year, with young children and other vulnerable populations facing higher health risks (World Health Organization, 2026). These concerns highlight the continuing importance of microbial contamination control in food safety.

In addition to planktonic bacterial contamination, biofilm formation by foodborne pathogens in food processing environments is another important cause of persistent contamination. Biofilms are complex microbial communities formed when bacteria adhere to food contact surfaces and become embedded in extracellular polymeric substances, which can enhance their tolerance to desiccation, cleaning procedures, disinfectants, and antimicrobial agents [6]. In the food industry, biofilms may develop on stainless steel equipment, conveyor belts, pipelines, cutting tools, and packaging materials, thereby serving as reservoirs for long-term bacterial persistence and cross-contamination. Previous studies have suggested that bacteria in mature biofilms are generally more difficult to eradicate than planktonic cells, and conventional cleaning and disinfection strategies may be insufficient to completely disrupt established biofilm structures [3]. Therefore, the development of safe, effective, and food-compatible natural antimicrobial agents is important for reducing microbial contamination risks, extending shelf life, and improving food safety.

Chemical preservatives and disinfectants are widely used in the food industry to control microbial contamination. However, their long-term or inappropriate use may raise concerns regarding consumer acceptance, environmental pressure, and the potential development of microbial tolerance. With increasing consumer demand for natural, clean label, and minimally processed foods, plant-derived antimicrobial agents have attracted growing attention in food preservation. Plant essential oils are volatile natural products formed during plant secondary metabolism and usually contain diverse bioactive compounds, including terpenes, alcohols, aldehydes, phenols, ketones, and esters [7,8]. Owing to their hydrophobic nature and biological activity, essential oils may exert antibacterial effects through multiple mechanisms, such as disrupting cell membrane structure, altering membrane permeability, causing leakage of intracellular constituents, affecting membrane potential, interfering with energy metabolism, and inhibiting biofilm formation [9,10]. Compared with single-target antimicrobial agents, the multi-component and multi-target characteristics of essential oils may provide advantages for their development as natural food antimicrobial and preservative agents.

In recent years, increasing attention has been paid to the inhibitory effects of essential oils and their major active components against foodborne pathogens. Our previous studies mainly focused on the chemical characterization and functional evaluation of citrus essential oils, and showed that different citrus essential oils possessed diverse volatile profiles as well as multiple biological activities, including antibacterial and antioxidant effects. In particular, several citrus essential oils showed inhibitory effects against different foodborne pathogens, suggesting their potential as natural food antimicrobial agents [11,12]. Beyond citrus essential oils, the antibacterial potential of essential oils from medicinal and aromatic plants has also attracted increasing interest. Traditional Chinese medicinal plants represent an abundant resource, and many medicinal or edible plants have long been used in foods, medicines, and health-related products. Plants such as *Lonicera japonica*, *Scutellaria baicalensis*, *Perilla frutescens*, *Artemisia argyi*, *Glycyrrhiza uralensis*, *Lycium barbarum*, *Poria cocos*, *Pueraria lobata*, and *Sinomenium acutum* have a long history of use, and their non-volatile extracts have been reported to possess antibacterial, antioxidant, or anti-inflammatory activities [13,14,15,16,17]. These nine plants were selected based on their traditional medicinal uses, reported biological activities, regional availability, relevance to our previous research, and potential for the comprehensive utilization of plant resources. Several species, including *S. acutum*, *L. japonica*, *P. cocos*, and *A. argyi*, are also important medicinal plants investigated by our team. However, systematic comparative information on the volatile composition and antibacterial activity of their essential oils remains limited.

Previous studies have reported that essential oils from *A. argyi*, *L. japonica*, *P. frutescens*, and other plant sources may possess antibacterial or antifungal activities [16,18,19,20]. Among these plants, *L. japonica* is a traditional medicinal plant commonly used for clearing heat and detoxification in traditional Chinese medicine, and it has also been used in herbal tea and functional food development [21,22]. Its flower buds are rich in bioactive constituents such as chlorogenic acid, flavonoids, and phenolic acids, making it an important source of plant-derived functional compounds [23]. Nevertheless, compared with non-volatile extracts of *L. japonica*, the role of *L. japonica* essential oil and other TCM herb essential oils in controlling foodborne pathogens remains insufficiently explored. Moreover, most available studies focus on a single essential oil or a single bacterial strain, whereas comparative evaluation of multiple TCM herb essential oils under the same experimental system, together with volatile composition analysis, mature biofilm removal assessment, and bacterial cell morphology observation, is still limited. Therefore, the systematic side-by-side comparison of multiple TCM herbal essential oils performed in this study under identical experimental conditions, together with the clarification of the relationship between volatile compounds and antibacterial activity, is valuable for the development of natural food antimicrobial agents.

Based on these considerations, this study selected nine TCM herb essential oils and systematically evaluated their antibacterial activities against *S. aureus*, *L. monocytogenes*, *E. coli*, and *S. paratyphi* B. Their ability to remove mature bacterial biofilms was also investigated. Gas chromatography–mass spectrometry was used to analyze the volatile composition of different essential oils, and principal component analysis, hierarchical clustering, and correlation analysis were performed to explore the potential relationship between major volatile compounds and antibacterial activity. In addition, *L. japonica* essential oil, which showed relatively strong overall antibacterial activity, was selected for bacterial growth curve analysis and scanning electron microscopy observation to further evaluate its effects on bacterial growth and cell surface morphology. This study aimed to screen TCM herb essential oils with potential application value in food antimicrobial control and to provide a basis for their future development as natural food antimicrobial and preservative candidates.

## 2. Materials and Methods

### 2.1. Microorganisms

Four common foodborne pathogens were used in this study: *Staphylococcus aureus* ATCC 29213, *Listeria monocytogenes* ATCC 19115, *Escherichia coli* ATCC 25922, and *Salmonella paratyphi* B CMCC 50094. All strains were stored at −80 °C in glycerol stocks and activated twice before use. Unless otherwise specified, the bacterial strains were cultured in Luria–Bertani broth (LB) at 37 °C.

### 2.2. TCM Herb Essential Oils

Nine TCM herb essential oils were selected in this study: *Sinomenium acutum* essential oil (SAEO), *Lonicera japonica* essential oil (LJEO), *Scutellaria baicalensis* essential oil (SBEO), *Pueraria lobata* essential oil (PLEO), *Glycyrrhiza uralensis* essential oil (GUEO), *Lycium barbarum* essential oil (LBEO), *Perilla frutescens* essential oil (PFEO), *Poria cocos* essential oil (PCEO), and *Artemisia argyi* essential oil (AAEO). All essential oils were purchased from Ji’an Huashuo Spice Oil Co., Ltd. (Jiangxi, China). The oils were stored in sealed amber glass bottles at 4 °C in the dark. Before use, the samples were equilibrated to room temperature and thoroughly mixed. Tween 20 was used as an emulsifier to improve the dispersion of essential oils in aqueous media, and the same concentration of Tween 20 was included in the control groups.

### 2.3. Gas Chromatography–Mass Spectrometry Analysis

The volatile composition of the nine TCM herb essential oils was analyzed by gas chromatography–mass spectrometry (GC–MS) according to our previous method with minor modifications [12]. Briefly, the essential oil samples were diluted tenfold with chromatographic-grade ethanol and filtered through a 0.22 μm organic membrane filter before injection. GC–MS analysis was performed using an Agilent GC–MS system equipped with an HP-5MS capillary column (15 m × 0.25 mm × 0.25 μm, HP-5 MS, Agilent). Helium was used as the carrier gas at a flow rate of 1.0 mL/min. The injector temperature was set at 250 °C, and the samples were analyzed in split injection mode with a split ratio of 1:20. The oven temperature program was as follows: the initial temperature was held at 70 °C for 1 min, increased to 150 °C at 3 °C/min and held for 2 min, then increased to 220 °C at 2 °C/min and held for 1 min, and finally increased to 250 °C and held for 20 min. The mass spectrometer was operated in electron impact ionization mode at 70 eV. To ensure analytical reproducibility, each sample was injected in triplicate.

Volatile compounds were initially identified by comparing their mass spectra with those in the National Institute of Standards and Technology mass spectral library (NIST 17). Retention indices (RIs) were further used to support compound identification. The RIs were calculated by linear interpolation using the retention times of a homologous series of n-alkanes analyzed under the same chromatographic conditions. The relative content of each volatile compound was determined by the peak area normalization method and expressed as the percentage of its peak area relative to the total peak area of all identified compounds.

### 2.4. Agar Diffusion Assay

The antibacterial activities of the nine TCM herb essential oils and selected individual compounds were evaluated using the agar diffusion method with minor modifications from a previously reported protocol [12]. Briefly, agar medium was poured into sterile Petri dishes with a diameter of 90 mm and allowed to solidify. Then, 100 μL of bacterial suspension at approximately 1 × 10^6^ CFU/mL was evenly spread onto the agar surface. Sterile paper disks with a diameter of 6 mm were placed on the inoculated agar plates, and each disk was loaded with the corresponding essential oil sample. Plates were incubated at 37 °C for 24 h. The antibacterial activity was evaluated by measuring the diameter of the inhibition zone (DIZ), including the 6 mm disk diameter. Results were expressed as the mean ± standard deviation of three inhibition zones. The antibacterial activity of selected individual compounds was determined using the same procedure.

### 2.5. Minimum Inhibitory Concentration Determination

The minimum inhibitory concentration (MIC) was determined using a modified broth microdilution method [24]. Briefly, bacterial cells were cultured to the logarithmic growth phase and adjusted to the required concentration. Essential oils were serially twofold diluted in culture medium in 96-well plates, with nine final concentrations ranging from 10% to 0.0390625% (*v*/*v*). The final volume in each well was 200 μL. Wells containing bacterial suspension without essential oils were used as growth controls, while wells containing medium without bacteria were used as blank controls. The bacterial suspension was added to each well to obtain a final bacterial concentration of approximately 1 × 10^6^ CFU/mL. Each treatment was performed in triplicate. After incubation at 37 °C for 24 h, resazurin solution was added to each well as a growth indicator. The MIC was defined as the lowest concentration of essential oil at which no visible bacterial growth was observed.

### 2.6. Biofilm Removal Assay

The biofilm removal assay was performed according to our previously established method with minor modifications [12]. Sterile glass coverslips or sterile stainless steel coupons (12 mm in diameter) were placed into the wells of 24-well plates. The four bacterial strains were cultured to the logarithmic growth phase and diluted with LB medium to approximately 1 × 10^6^ CFU/mL. Then, 2 mL of bacterial suspension was added to each well, and the plates were statically incubated at 37 °C for 48 h to allow mature biofilm formation.

After incubation, the culture medium was removed, and fresh medium containing 1.25% essential oil was added to the treatment groups. The control group received the same volume of medium without essential oil but containing the same concentration of emulsifier. The plates were incubated at 37 °C for another 6 h. After treatment, the wells were washed three times with phosphate-buffered saline (PBS) to remove planktonic cells and residual essential oil. Subsequently, 1% crystal violet solution was added to each well and incubated at room temperature for 20 min. After staining, the wells were washed three times with PBS to remove excess dye. The coverslips were then removed, air-dried, and photographed.

Finally, the coverslips were returned to the wells, and 2 mL of absolute ethanol was added to dissolve the crystal violet bound to the biofilms. The absorbance was measured at 600 nm using a microplate reader (Thermo Fisher Scientific, Waltham, MA, USA). The biofilm removal rate was calculated using the following equation:

$$Biofilm\;removal\;rate\left(\%\right)=\left[\left(A_{control}-A_{treatment}\right)/A_{control}\right]\times 100$$

where *A_control_* represents the absorbance of the control group, and *A_treatment_* represents the absorbance of the essential oil-treated group.

### 2.7. Effect of LJEO on Bacterial Growth Curves

Based on the results of the DIZ and MIC assays, LJEO was selected for further evaluation of its effects on bacterial growth kinetics [2]. The four test strains were cultured to the logarithmic growth phase and diluted to approximately 1 × 10^6^ CFU/mL. LJEO was added to the bacterial suspensions to obtain final concentrations of MIC, 1/2 MIC, 1/4 MIC, and 1/8 MIC. Bacterial suspensions without LJEO but containing the same concentration of emulsifier were used as controls. The cultures were incubated at 37 °C, and the optical density at 600 nm (OD_600_) was measured at 0, 2, 4, 6, 8, 10, and 12 h. Growth curves were generated by plotting incubation time against OD_600_. Each experiment was performed in triplicate.

### 2.8. Scanning Electron Microscopy Assay

Scanning electron microscopy (SEM) was used to observe bacterial cell morphology according to a previously reported method with minor modifications [2]. The four bacterial strains were separately inoculated into LB medium and cultured overnight at 37 °C. The bacterial suspensions were then adjusted to approximately 1 × 10^8^ CFU/mL. LJEO was added to the bacterial suspensions at 1 × MIC, while bacterial suspensions without LJEO were used as controls. After incubation at 37 °C for 1 h, the cells were collected by centrifugation for 10 min and washed three times with PBS to remove residual medium components.

The collected bacterial cells were fixed with 2.5% glutaraldehyde. After fixation, the samples were washed three times with PBS and dehydrated using a graded ethanol series of 10%, 30%, 50%, 70%, and 90%, followed by two rounds of dehydration with 100% ethanol. All dehydration steps were performed at room temperature. The dehydrated samples were freeze-dried, sputter-coated with gold, and observed using a scanning electron microscope (SU8010; Hitachi High-Tech Corporation, Tokyo, Japan).

### 2.9. Statistical Analysis

GC–MS data were processed using MSD ChemStation software (Agilent Technologies, Santa Clara, CA, USA; https://www.agilent.com/, accessed on 28 July 2026). Graphs were generated using GraphPad Prism 9 (GraphPad Software, San Diego, CA, USA). Heatmaps and UpSet plots were generated using the ChiPlot online platform (https://www.chiplot.online/ accessed on 28 July 2026). For multivariate and correlation analyses, compounds not detected in a given sample were assigned a value of 0. Pearson or Spearman correlation analysis was used according to the data distribution to evaluate the relationship between volatile compounds and antibacterial activity.

## 3. Results

### 3.1. DIZ of TCM Herb Essential Oils Against Foodborne Pathogens

**Figure 1:**
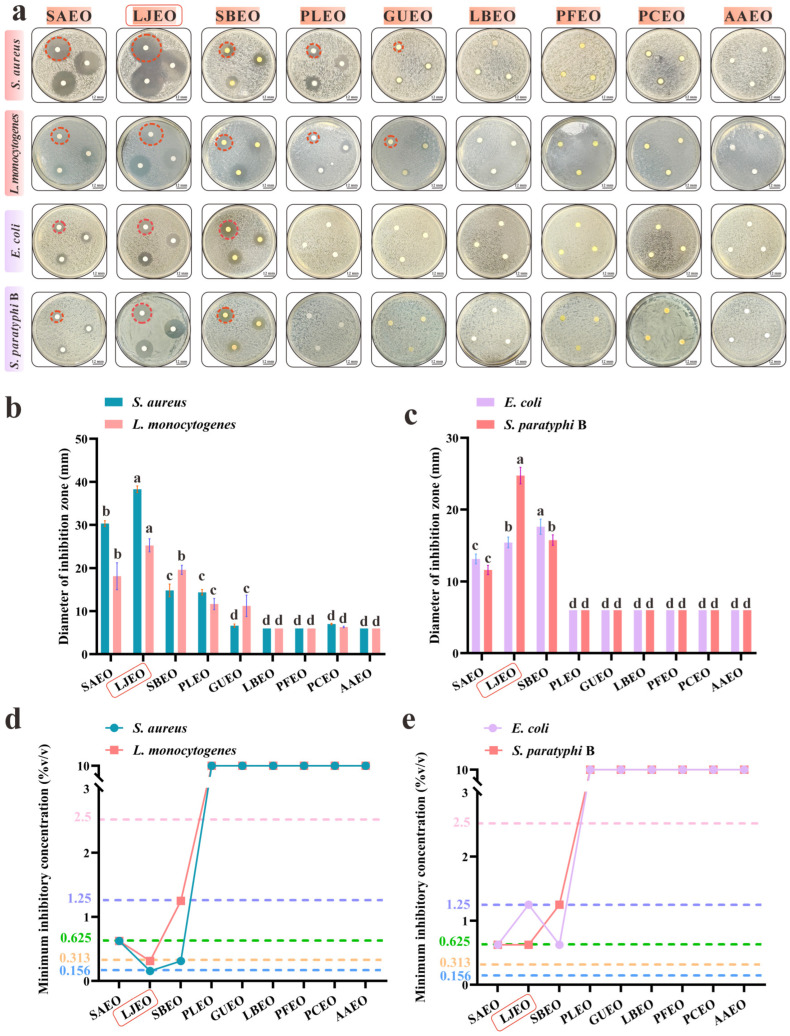
Antibacterial activities of nine TCM herb essential oils against four foodborne pathogens. (**a**) DIZ results of the nine TCM herb essential oils against the four tested bacteria; (**b**) DIZ results of the nine TCM herb essential oils against the Gram-positive bacteria *S. aureus* and *L. monocytogenes*; (**c**) DIZ results of the nine TCM herb essential oils against the Gram-negative bacteria *E. coli* and *S. paratyphi* B; (**d**) MIC results of the nine TCM herb essential oils against the Gram-positive bacteria; (**e**) MIC results of the nine TCM herb essential oils against the Gram-negative bacteria. The circles represent the inhibition zones produced by the essential oils in the agar diffusion assay. The boxes highlight the antibacterial activity of LJEO, which was selected for subsequent mechanistic and biofilm studies. For panels (**b**,**c**), data are presented as the mean ± standard deviation (SD) of three independent experiments. Different lowercase letters above the bars for the same bacterial strain indicate significant differences among the essential oils according to one-way ANOVA followed by Tukey’s multiple-comparison test (*p* < 0.05).

The agar diffusion assay was used to preliminarily evaluate the inhibitory effects of the nine TCM herb essential oils against four foodborne pathogens. As shown in Figure 1a–c, the tested essential oils exhibited distinct inhibitory effects against different bacterial strains. Among them, LJEO showed the most notable overall antibacterial activity under the present experimental conditions.

LJEO exhibited the largest inhibition zone against *Staphylococcus aureus*, with a DIZ of 38.29 ± 0.79 mm, which was higher than that observed for the other essential oils. LJEO also showed clear inhibitory effects against *Listeria monocytogenes* and *Salmonella paratyphi* B, with DIZ values of 25.29 ± 1.51 mm and 24.75 ± 1.15 mm, respectively. In comparison, the inhibitory effect of LJEO against *Escherichia coli* was relatively weaker, although a visible inhibition zone was still observed, with a DIZ of 15.43 ± 0.73 mm.

In addition to LJEO, SAEO and SBEO also showed measurable antibacterial activity, although their antibacterial spectra differed from that of LJEO. SAEO exhibited a relatively strong inhibitory effect against *S. aureus*, with a DIZ of 30.31 ± 0.68 mm, while its inhibitory effects against the other three strains were weaker. SBEO showed moderate inhibitory activity against all four tested strains, with relatively large inhibition zones against *L. monocytogenes* and *E. coli*, reaching 19.61 ± 1.06 mm and 17.62 ± 1.06 mm, respectively. In contrast, PLEO, GUEO, and PCEO only showed weak inhibitory effects against some bacterial strains, whereas the inhibition zone diameters of LBEO, PFEO, and AAEO were close to the diameter of the paper disk, indicating that no obvious inhibitory activity was observed under the present test conditions.

Overall, the DIZ results showed that, among the nine commercial TCM herb essential oils tested in this study, LJEO exhibited relatively prominent antibacterial activity, particularly against *S. aureus*, *L. monocytogenes*, and *S. paratyphi* B.

### 3.2. MIC of TCM Herb Essential Oils Against Foodborne Pathogens

The MICs of the nine TCM herb essential oils against the four foodborne pathogens were further determined using the broth microdilution method. As shown in Figure 1d,e, the MIC values differed markedly among the tested essential oils. LJEO, SAEO, and SBEO showed relatively stronger antibacterial activity, whereas PLEO, GUEO, LBEO, PFEO, PCEO, and AAEO all exhibited MIC values of 10%, suggesting weaker inhibitory efficacy within the tested concentration range.

LJEO showed the lowest MIC against *S. aureus*, with a value of 0.15625%, indicating a strong inhibitory effect against this strain. The MIC of LJEO against *L. monocytogenes* was 0.3125%, also suggesting relatively high antibacterial activity. In contrast, the MIC values of LJEO against *S. paratyphi* B and *E. coli* were 0.625% and 1.25%, respectively, indicating that these two Gram-negative bacteria were less sensitive to LJEO than *S. aureus* and *L. monocytogenes*, especially *E. coli*.

SAEO showed an MIC value of 0.625% against all four tested strains, suggesting relatively balanced broad-spectrum inhibitory activity. SBEO showed MIC values of 0.3125% against *S. aureus* and 0.625% against *E. coli*, whereas its MIC values against *L. monocytogenes* and *S. paratyphi* B were both 1.25%. Taken together, the MIC results showed that LJEO exhibited relatively strong inhibitory activity against *S. aureus* and *L. monocytogenes*, while SAEO and SBEO also displayed inhibitory effects against specific bacterial strains.

When the DIZ and MIC results were considered together, LJEO showed favorable overall antibacterial performance, especially against *S. aureus* and *L. monocytogenes*.

### 3.3. Biofilm Removal Activity

**Figure 2:**
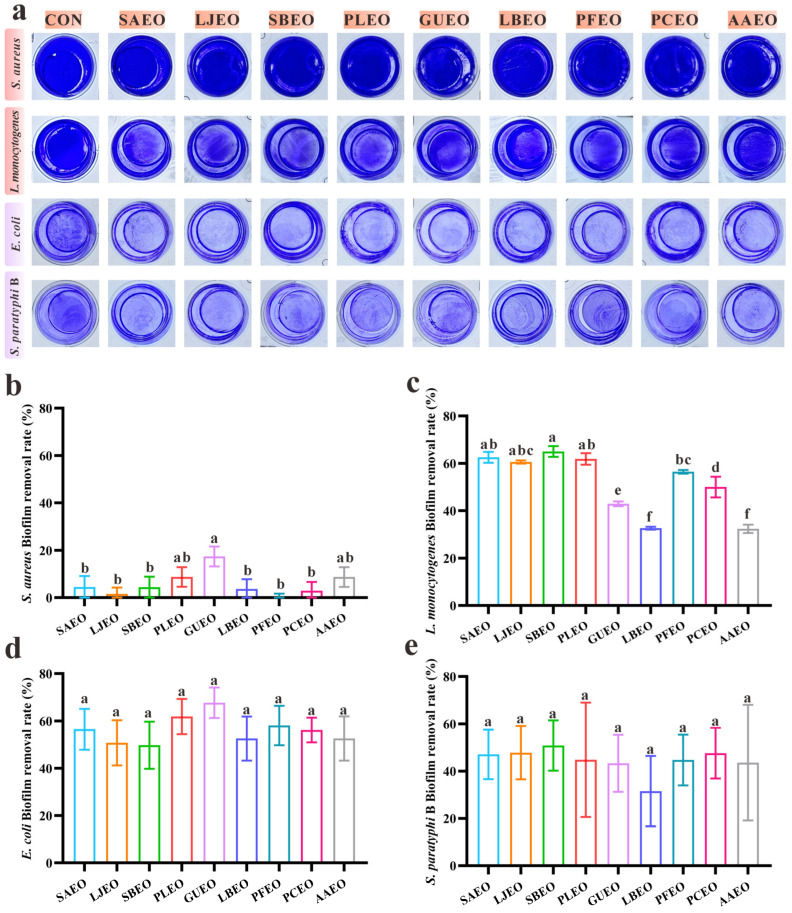
Removal effects of nine TCM herb essential oils on mature bacterial biofilms. (**a**) Crystal violet staining results of mature biofilms formed by the four tested bacteria after treatment with the nine TCM herb essential oils; (**b**) removal rates of the nine TCM herb essential oils against mature *L. monocytogenes* biofilms; (**c**) removal rates of the nine TCM herb essential oils against mature *S. aureus* biofilms; (**d**) removal rates of the nine TCM herb essential oils against mature *E. coli* biofilms; (**e**) removal rates of the nine TCM herb essential oils against mature *S. paratyphi* B biofilms. For panels (**b**–**e**), data are presented as the mean ± standard deviation (SD) of three independent experiments. Different lowercase letters above the bars indicate significant differences among the essential oil treatments according to one-way ANOVA followed by Tukey’s multiple-comparison test (*p* < 0.05).

Given the importance of biofilms in foodborne microbial contamination, the crystal violet staining method was used to evaluate the ability of the nine TCM herb essential oils to remove mature biofilms formed by the four foodborne pathogens. As shown in Figure 2, mature biofilms formed by different bacterial species responded differently to essential oil treatment, indicating that the biofilm removal effect was strongly species-dependent. Overall, the biofilms of *L. monocytogenes*, *E. coli*, and *S. paratyphi* B were more responsive to some essential oil treatments, whereas *S. aureus* biofilms showed greater tolerance to all tested essential oils. For *L. monocytogenes* biofilms, most essential oils exhibited some degree of removal activity. Among them, SBEO, SAEO, PLEO, and LJEO showed relatively high removal rates of 65.02 ± 2.26%, 62.56 ± 2.27%, 61.87 ± 2.43%, and 60.58 ± 0.70%, respectively. Although LJEO did not show the highest removal rate in this assay, its removal rate remained above 60% with relatively small variation among replicates, indicating a stable removal effect against mature *L. monocytogenes* biofilms. PFEO and PCEO showed moderate biofilm removal effects, whereas GUEO, LBEO, and AAEO showed relatively weak activity.

In contrast, none of the nine essential oils exhibited obvious removal activity against mature *S. aureus* biofilms. The overall removal rates were low, with the highest value observed for GUEO at only 17.45 ± 4.19%, while most other essential oil treatments showed removal rates below 10%. The removal rate of LJEO against *S. aureus* biofilms was only 1.57 ± 2.71%, indicating that although LJEO strongly inhibited planktonic *S. aureus*, it was not effective in removing mature *S. aureus* biofilms under the present conditions.

For *E. coli* biofilms, the tested essential oils generally showed better removal effects. GUEO exhibited the highest removal rate of 67.70 ± 6.42%, followed by PLEO, PFEO, SAEO, and PCEO, with removal rates of 61.88 ± 7.45%, 58.08 ± 8.35%, 56.52 ± 8.63%, and 56.20 ± 5.23%, respectively. LJEO also showed a measurable removal effect against *E. coli* biofilms, with a removal rate of 50.80 ± 9.55%. For *S. paratyphi* B biofilms, the removal effects of the essential oils were generally moderate. SBEO, LJEO, PCEO, and SAEO showed relatively higher removal rates of 50.86 ± 10.64%, 47.82 ± 11.31%, 47.60 ± 10.71%, and 47.10 ± 10.47%, respectively.

Overall, the biofilm removal assay indicated that the ability of TCM herb essential oils to remove mature biofilms was not fully consistent with their inhibitory effects against planktonic cells. LJEO showed different degrees of removal activity against *L. monocytogenes*, *E. coli*, and *S. paratyphi* B biofilms, but showed little effect on mature *S. aureus* biofilms.

### 3.4. Volatile Composition and Clustering Analysis of TCM Herb Essential Oils

To further clarify the possible chemical basis underlying the differences in antibacterial activity among the essential oils, GC–MS was used to analyze the volatile composition of the nine TCM herb essential oils. The qualitative identification results are shown in Table 1, and the quantitative results obtained by peak area normalization are shown in Table 2. A total of 156 volatile compounds were identified across the nine essential oils and classified into 10 major chemical categories.

**Figure 3:**
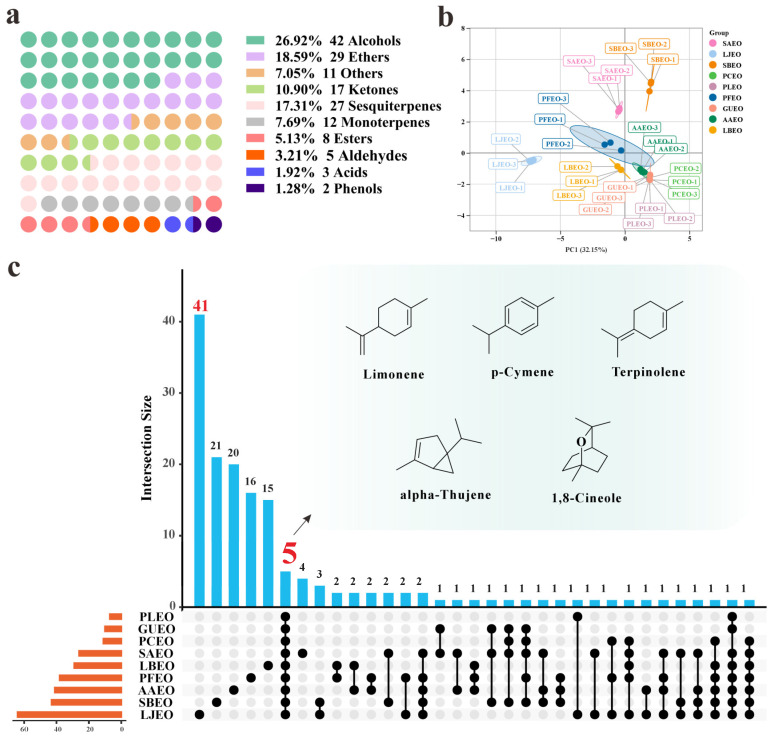
Volatile composition and overall variation analysis of the nine TCM herb essential oils. (**a**) Distribution of volatile compound classes and the number of identified compounds in the nine TCM herb essential oils; (**b**) principal component analysis (PCA) based on the relative contents of volatile compounds; (**c**) UpSet analysis of shared and unique volatile compounds among the nine TCM herb essential oils.

As shown in Figure 3a, based on the number of identified compounds, alcohols represented the largest category, with 42 compounds accounting for 26.92% of all identified compounds. Ethers were the second-largest category, with 29 compounds accounting for 18.59%. Other compounds, ketones, and sesquiterpenes accounted for 16.03%, 10.90%, and 8.33%, respectively. Monoterpenes, esters, aldehydes, acids, and phenols showed lower proportions, accounting for 7.69%, 5.13%, 3.21%, 1.92%, and 1.28%, respectively. These results indicated that the nine TCM herb essential oils contained diverse types of volatile compounds, with alcohols and ethers being the dominant categories in terms of compound number. Principal component analysis based on the relative abundance of volatile compounds further showed clear differences in the overall chemical composition among the essential oil samples. As shown in Figure 3b, PC1 and PC2 explained 32.15% and 16.90% of the total variance, respectively. LJEO was clearly separated from the other essential oils along PC1, suggesting that its volatile composition was distinct from those of the other samples. The UpSet analysis further supported this observation. As shown in Figure 3c, LJEO contained the highest number of unique compounds, with 41 compounds detected only in LJEO. SBEO and AAEO contained 21 and 20 unique compounds, respectively, while PFEO and LBEO each contained 15 unique compounds. In addition, five compounds were shared by all nine essential oils, namely α-thujene, limonene, p-cymene, terpinolene, and 1,8-cineole, suggesting that these terpene-related compounds were common volatile constituents among the tested TCM herb essential oils. The quantitative results in Table 2 showed that the major volatile compounds with relatively high abundance in LJEO included linalool (10.30%), p-cymene (6.72%), borneol (6.44%), 1,4-cineole (6.10%), 1,8-cineole (5.90%), limonene (5.88%), menthone (4.71%), isoborneol (4.43%), and α-thujene (4.26%). These compounds suggested that LJEO possessed a complex volatile profile characterized mainly by terpenes and oxygenated terpene derivatives.

**Figure 4:**
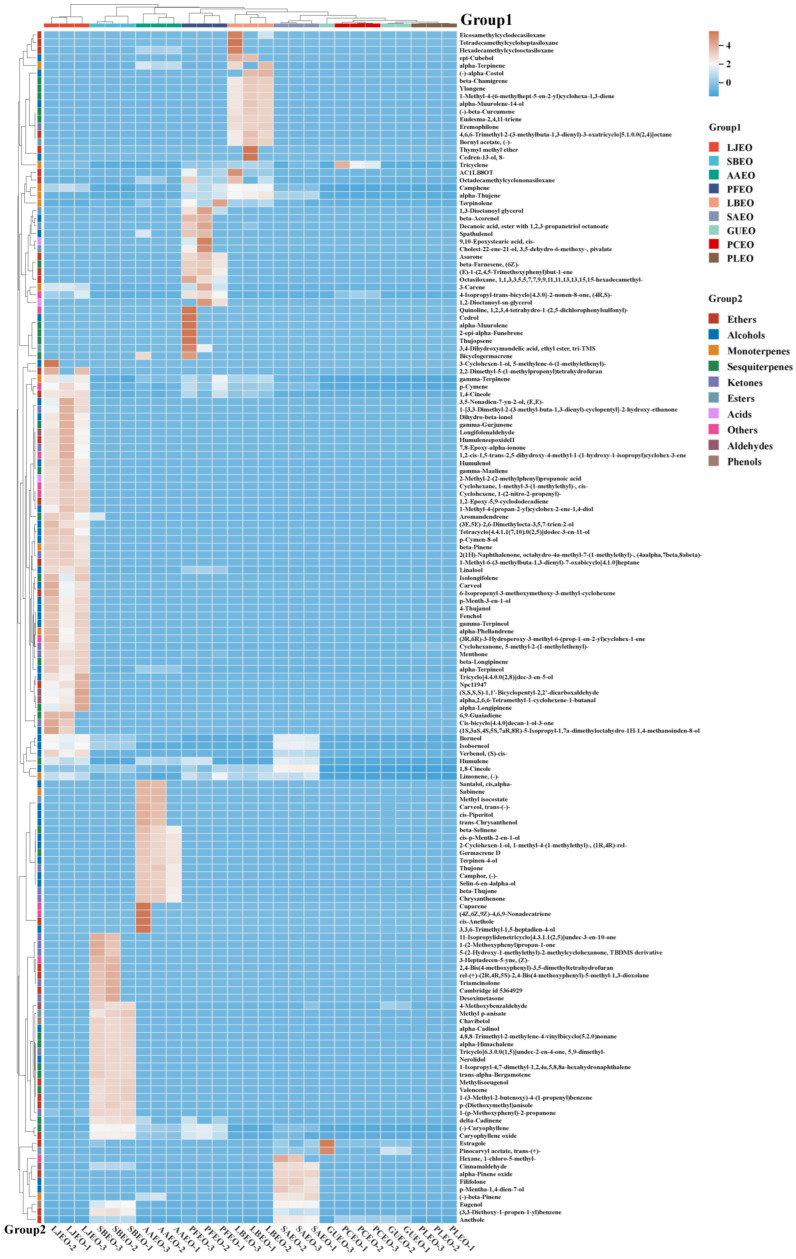
Hierarchical clustering heatmap of volatile compounds in the nine TCM herb essential oils.

To further visualize the abundance distribution of individual compounds among different essential oil samples, a hierarchical clustering heatmap was generated based on the relative abundance of volatile compounds. As shown in Figure 4, replicates of the same essential oil generally clustered together, indicating good repeatability of the GC–MS analysis. Different essential oils formed relatively independent clustering patterns, reflecting distinct chemical fingerprints among the samples. LJEO showed a relatively independent abundance distribution pattern in the heatmap, with enrichment of several terpenes and oxygenated terpene compounds. This result was consistent with the PCA and UpSet analyses and further confirmed the unique volatile composition of LJEO compared with the other essential oils.

### 3.5. Major Volatile Compounds and Their Correlation with Antibacterial Activity

**Figure 5:**
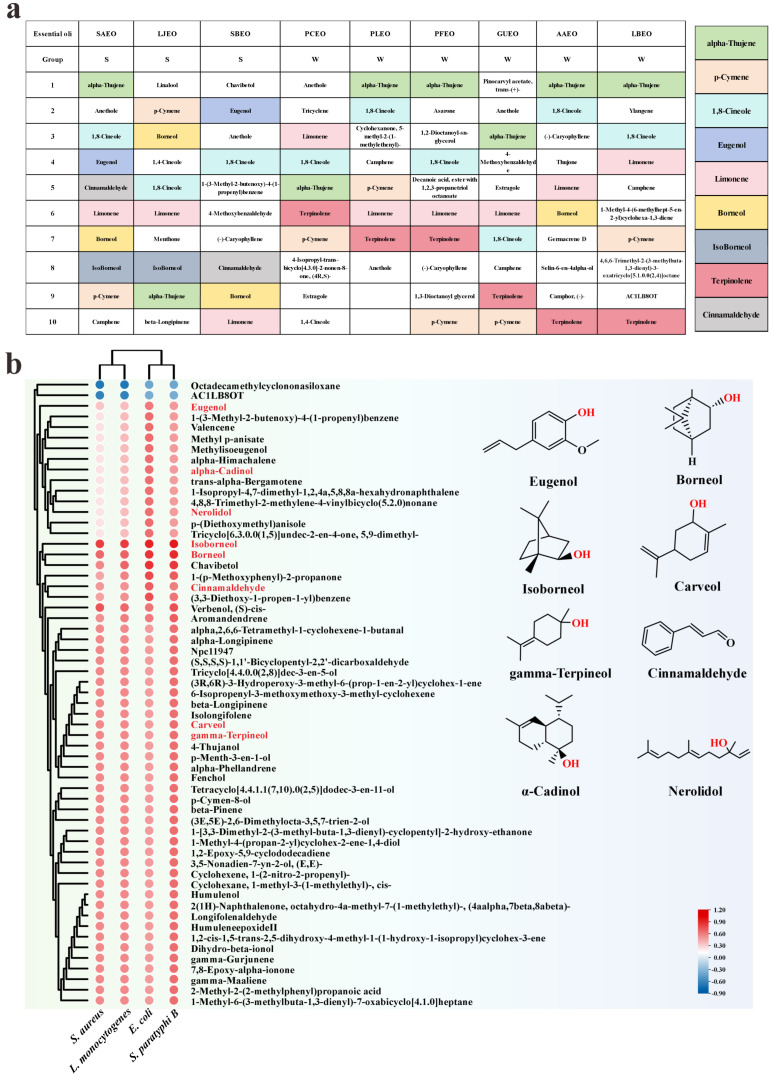
Major volatile compounds of the nine TCM herb essential oils and their correlation with antibacterial activity. (**a**) Distribution of the top ten major volatile compounds with the highest relative contents in the nine TCM herb essential oils; (**b**) correlation heatmap between the relative contents of volatile compounds and the diameters of inhibition zones against the four tested bacteria.

To further explore the relationship between volatile composition and antibacterial activity, the top ten compounds with the highest relative abundance in each essential oil were summarized. As shown in Figure 5a, based on the overall antibacterial performance, SAEO, LJEO, and SBEO were classified as the relatively active group, and their major compound profiles differed from those of the weakly active essential oils.

The major characteristic compounds of SAEO included α-thujene, anethole, 1,8-cineole, eugenol, cinnamaldehyde, limonene, borneol, and isoborneol. The major compounds in LJEO included linalool, p-cymene, borneol, 1,4-cineole, 1,8-cineole, limonene, menthone, isoborneol, and α-thujene. SBEO was mainly characterized by chavibetol, eugenol, anethole, 1,8-cineole, 4-methoxybenzaldehyde, caryophyllene, cinnamaldehyde, borneol, and limonene. Notably, compounds such as eugenol, cinnamaldehyde, borneol, isoborneol, limonene, and 1,8-cineole were relatively prominent in the active essential oils. Correlation analysis was then performed to evaluate the association between volatile compounds and antibacterial activity. As shown in Figure 5b, different compounds showed varying degrees of correlation with antibacterial activity. Among them, eugenol, borneol, isoborneol, cinnamaldehyde, carveol, γ-terpineol, α-cadinol, and nerolidol exhibited clear positive correlations with antibacterial activity and appeared as strong positive signals in the correlation heatmap. These compounds mainly belonged to phenols, aldehydes, alcohols, and terpene derivatives, suggesting that they may be associated with the differences in antibacterial activity among the essential oils.

The positive correlations of these compounds were not limited to a single bacterial strain but were associated with the inhibitory effects against multiple tested strains. This result suggested that the antibacterial activity of TCM herb essential oils may not be determined by a single dominant compound, but rather by the combined contribution of multiple active constituents. In particular, the relative enrichment of these positively correlated compounds in SAEO, LJEO, and SBEO was generally consistent with their larger inhibition zones and lower MIC values.

### 3.6. Effects of LJEO on Bacterial Growth Kinetics

**Figure 6:**
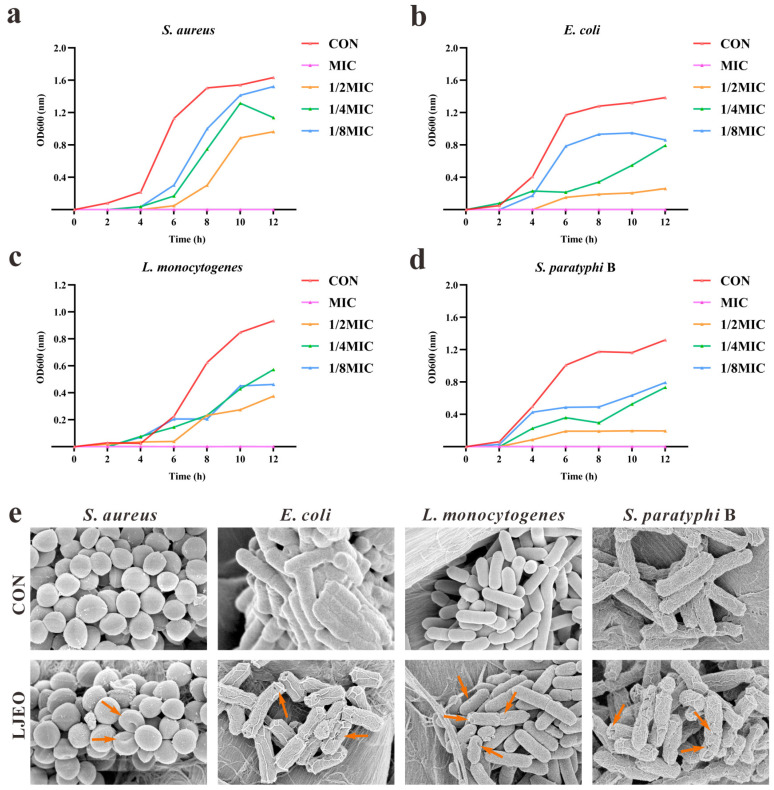
Effects of LJEO on bacterial growth kinetics and cell morphology. (**a**) Effects of LJEO on the growth curve of *S. aureus*; (**b**) effects of LJEO on the growth curve of *E. coli*; (**c**) effects of LJEO on the growth curve of *L. monocytogenes*; (**d**) effects of LJEO on the growth curve of *S. paratyphi* B; (**e**) scanning electron microscopy observation of morphological changes in the four tested bacteria before and after LJEO treatment.

Based on the antibacterial screening results, LJEO was selected to further evaluate its effects on the growth kinetics of the four tested strains. As shown in Figure 6a–d, the OD_600_ values of *S. aureus*, *E. coli*, *L. monocytogenes*, and *S. paratyphi* B in the control groups increased rapidly with incubation time, indicating normal bacterial growth. In contrast, LJEO at the MIC markedly suppressed the growth of all four bacterial strains within 12 h, with OD_600_ values remaining at low levels throughout the incubation period.

At sub-inhibitory concentrations, LJEO still delayed bacterial growth, although the response varied among bacterial strains. Treatment with 1/2 MIC LJEO showed an obvious growth inhibitory effect against all four bacteria, particularly against *E. coli*, *L. monocytogenes*, and *S. paratyphi* B. When the LJEO concentration was reduced to 1/4 MIC and 1/8 MIC, the growth of the tested strains gradually recovered, but the OD_600_ values remained generally lower than those of the control group. Compared with the other strains, *S. aureus* recovered more rapidly under sub-inhibitory LJEO treatment, whereas *E. coli*, *L. monocytogenes*, and *S. paratyphi* B appeared more sensitive to LJEO exposure in the growth curve assay. These results indicated that LJEO inhibited bacterial growth in a concentration-dependent manner, with the MIC treatment showing the most stable inhibitory effect.

### 3.7. Effects of LJEO on Bacterial Cell Morphology

Scanning electron microscopy was used to observe changes in bacterial cell morphology after LJEO treatment. As shown in Figure 6e, untreated control cells exhibited intact morphology. *S. aureus* cells showed a regular spherical shape with a relatively smooth surface, while *E. coli*, *L. monocytogenes*, and *S. paratyphi* B cells displayed typical rod-shaped morphology with clear cell boundaries and intact surface structures. After LJEO treatment, all four bacterial strains exhibited different degrees of morphological damage. *S. aureus* cells showed surface depressions, while *E. coli* cells displayed obvious shrinkage, collapse, and surface disruption. The surface of *L. monocytogenes* cells became depressed, and *S. paratyphi* B cells also showed clear shrinkage, collapse, and morphological distortion. These morphological changes indicated that LJEO treatment could damage bacterial cell surface structures, which was consistent with the inhibitory effects observed in the growth curve assay. These results suggested that LJEO may inhibit bacterial growth, at least in part, by affecting cell surface integrity.

### 3.8. Biofilm Removal on Stainless Steel Surfaces

**Figure 7:**
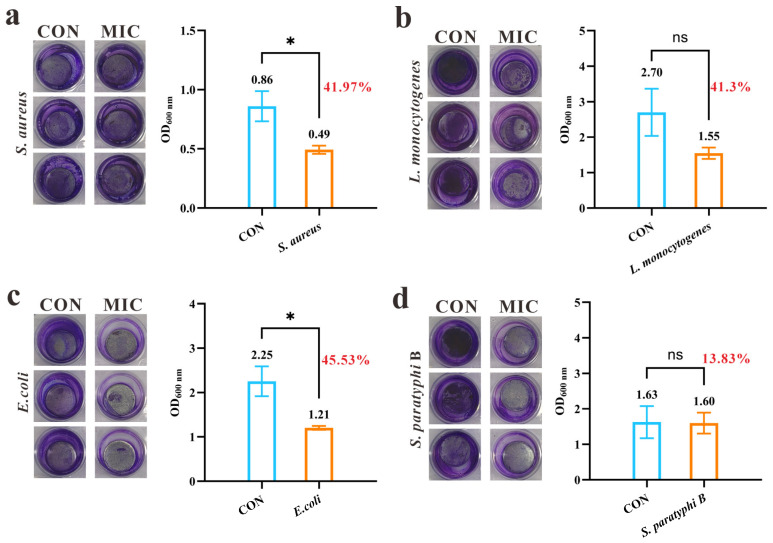
Biofilm removal activity of LJEO against mature biofilms formed on stainless steel coupons. Representative crystal violet-stained images and quantitative analysis of biofilm biomass for (**a**) *Staphylococcus aureus*, (**b**) *Listeria monocytogenes*, (**c**) *Escherichia coli*, and (**d**) *Salmonella paratyphi* B after treatment with LJEO at the MIC for 6 h. Biofilm biomass was quantified by measuring the absorbance at 600 nm after crystal violet solubilization. The red percentages indicate the biofilm removal rates relative to the untreated control (CON). Data are presented as mean ± SD (*n* = 3). * indicates *p* < 0.05 compared with the untreated control (CON); ns, not significant.

To further evaluate the practical antibiofilm potential of LJEO, its biofilm removal activity on stainless steel surfaces was assessed using crystal violet staining (Figure 7). Representative images showed that LJEO treatment visibly reduced biofilm formation on stainless steel coupons compared with the untreated control.

Quantitative analysis showed that LJEO significantly reduced the biofilm biomass of *S. aureus* and *E. coli* by 41.97% and 45.53%, respectively (*p* < 0.05). Biofilm biomass was also reduced in *L. monocytogenes* (41.30%) and *S. paratyphi* B (13.83%); however, these differences were not statistically significant (*p* > 0.05). Among the four tested bacteria, the greatest biofilm removal effect was observed against *E. coli*, whereas the weakest effect was observed against *S. paratyphi* B.

Overall, these results demonstrate that LJEO can effectively remove preformed biofilms from stainless steel surfaces, although its efficacy varies among different bacterial species.

## 4. Discussion

Foodborne pathogen contamination remains a major concern in food safety, particularly because microbial hazards may occur throughout food production, processing, storage, and distribution. At the same time, increasing consumer demand for natural preservatives and green preservation strategies has promoted the exploration of plant essential oils as natural antimicrobial agents. Essential oils are complex volatile mixtures containing terpenes, phenols, aldehydes, alcohols, and other bioactive compounds, which may inhibit microorganisms by disrupting cell membranes, altering membrane permeability, interfering with energy metabolism, or inhibiting biofilm development. Therefore, essential oils have been considered promising candidates for food antimicrobial preservation and microbial risk control [25]. In this context, the comparative screening of TCM herb essential oils may provide useful information for identifying new natural antimicrobial resources for foodborne pathogen control.

In the present study, nine commercial TCM herb essential oils were compared for their antibacterial effects against four representative foodborne pathogens. The results showed clear differences in antibacterial activity among the tested essential oils. Among them, LJEO showed the most notable overall inhibitory activity in the DIZ assay, particularly against *Staphylococcus aureus*, *Listeria monocytogenes*, and *Salmonella paratyphi* B. The MIC results further indicated that LJEO was more effective against *S. aureus* and *L. monocytogenes*, while a higher MIC was observed against *Escherichia coli*. These results are generally consistent with previous reports showing that *L. japonica* essential oil and extracts possess inhibitory effects against *L. monocytogenes*, *S. aureus*, *Salmonella* spp., and some *E. coli* strains, although bacterial sensitivity may vary among species and strains [26]. Our previous studies on citrus essential oils also showed that Gram-positive bacteria were generally more sensitive than Gram-negative bacteria to essential oil treatment [12]. This difference may be partly explained by the outer membrane of Gram-negative bacteria, in which the lipopolysaccharide layer can limit the penetration of hydrophobic antimicrobial compounds [27]. However, LJEO still showed clear inhibitory activity against *S. paratyphi* B, suggesting that the response of Gram-negative bacteria to the same essential oil is not uniform. Such differences may be related to variations in outer membrane structure, membrane lipid composition, efflux systems, metabolic state, and the specific intracellular targets affected by essential oil components.

The DIZ and MIC results were not completely consistent. For example, LJEO produced a relatively large inhibition zone against *S. paratyphi* B in the agar diffusion assay, but its MIC was not the lowest among the tested essential oils and was similar to that of SAEO. This observation indicates that antibacterial activity of essential oils should not be evaluated using a single method. The agar diffusion assay is influenced not only by antimicrobial potency but also by volatility, hydrophobicity, and diffusion capacity in agar medium, which may lead to an underestimation of true antibacterial activity for hydrophobic essential oils. Therefore, DIZ values should be interpreted with caution and are best used for relative comparisons rather than as absolute potency rankings. In contrast, MIC determination better reflects the inhibitory effect of essential oils in a liquid culture system [23]. Since plant essential oils are complex mixtures, the release, solubility, diffusion, and interaction of different components may vary across experimental systems. The combined interpretation of DIZ and MIC results thus provides a more comprehensive assessment of the antibacterial potential of TCM herb essential oils. Therefore, the combined interpretation of DIZ and MIC results provides a more comprehensive assessment of the antibacterial potential of TCM herb essential oils.

In addition to LJEO, SAEO and SBEO also showed antibacterial activity, but their antibacterial spectra were different from that of LJEO. *S. acutum* is traditionally known as a medicinal plant rich in sinomenine, an isoquinoline alkaloid with pharmacological activities, particularly in inflammation-related conditions [28]. *S. baicalensis* is also a widely used traditional medicinal plant and is known for its rich bioactive constituents, especially flavonoids [29]. However, compared with non-volatile extracts, the antibacterial potential of their essential oils remains less characterized. In this study, SAEO showed relatively stable MIC values against all four tested strains, suggesting a more balanced inhibitory spectrum, whereas SBEO showed better activity against *S. aureus* and *E. coli* than against *L. monocytogenes* and *S. paratyphi* B. These differences suggest that the antibacterial activity of TCM herb essential oils is not determined simply by the botanical origin of the plant, but is more closely associated with the specific volatile composition of the essential oil. In contrast, PLEO, GUEO, LBEO, PFEO, PCEO, and AAEO showed weaker antibacterial effects under the present experimental conditions. This does not necessarily mean that these plant species lack antimicrobial potential, as essential oil activity can be influenced by plant origin, extraction process, storage, batch differences, and chemical composition. Rather, these results highlight the importance of systematic screening to identify essential oil samples with practical application potential.

The GC–MS results provided a chemical basis for understanding the differences in antibacterial activity among the tested essential oils. A total of 156 volatile compounds were identified, and LJEO was clearly separated from the other oils in PCA and hierarchical clustering analyses. LJEO also contained the highest number of unique compounds, indicating a distinct volatile profile. The major components of LJEO included linalool, p-cymene, borneol, 1,4-cineole, 1,8-cineole, limonene, menthone, and isoborneol, most of which belong to terpenes or oxygenated terpene derivatives. Previous studies have reported the presence of active components such as nerolidol, linalool, p-cymene, and eugenol in *L. japonica* essential oil, and these compounds have been associated with antimicrobial activity [17,26]. In addition, recent studies on *L. japonica* extracts in meat preservation suggested that honeysuckle-derived bioactive compounds may help maintain refrigerated food quality and suppress microbial growth [30]. Therefore, the relatively strong antibacterial activity of LJEO observed in this study may be associated with its enrichment in oxygenated terpenes and aromatic volatile compounds.

Further comparison of SAEO, LJEO, and SBEO indicated that the antibacterial activities of these three relatively active essential oils may be supported by different chemical profiles. SAEO contained eugenol, cinnamaldehyde, 1,8-cineole, limonene, borneol, and isoborneol, among which eugenol and cinnamaldehyde are well-recognized antimicrobial constituents in plant essential oils. These compounds have been reported to disrupt membrane integrity, cause leakage of intracellular materials, and interfere with energy metabolism [31,32]. SBEO was also rich in eugenol, anethole, 1,8-cineole, cinnamaldehyde, and borneol, suggesting that phenolic and aldehyde compounds may contribute to its antibacterial activity. By contrast, LJEO was characterized by linalool, p-cymene, borneol, cineole derivatives, and limonene, which may be more closely associated with membrane perturbation and cell surface damage. In particular, linalool has been widely investigated for its antimicrobial activity against different microorganisms [23,24,33,34,35]. These findings suggest that the antibacterial effects of the three active essential oils are unlikely to arise from a single shared dominant compound, but may instead result from different combinations of volatile constituents.

Correlation analysis further identified several compounds that may be associated with antibacterial activity, including eugenol, borneol, isoborneol, cinnamaldehyde, carveol, γ-terpineol, α-cadinol, and nerolidol. These compounds include phenols, aldehydes, oxygenated monoterpenes, and sesquiterpene alcohols, all of which are commonly found in antimicrobial plant essential oils. Previous studies have shown that eugenol and cinnamaldehyde can exert antibacterial effects by disrupting bacterial membranes, affecting membrane potential, and inhibiting key enzymatic processes [31,32]. Linalool has also been shown to affect *E. coli* viability by regulating lipopolysaccharide synthesis and modification, ribosome assembly, and substrate transport-related protein [23]. Proteomic studies of *L. monocytogenes* exposed to linalool further suggested that linalool may affect multiple cellular targets, including the cell membrane, cell wall, nucleoid, and ribosome [35]. Nevertheless, the correlation analysis in this study only indicates statistical associations between specific volatile compounds and antibacterial activity, and does not prove that any single compound is the decisive antibacterial factor. Essential oils are complex mixtures, and their antibacterial effects often result from additive or synergistic interactions among multiple constituents rather than from the independent action of one abundant compound. Essential oils may also act through multiple targets, including membrane disruption, quorum sensing interference, biofilm inhibition, and efflux pump modulation, which may contribute to their potential as natural antimicrobial agents [36]. Future studies should therefore include antibacterial activity testing of individual compounds, component recombination assays, and fractional inhibitory concentration index analysis to clarify possible synergistic interactions.

The biofilm removal results also deserve attention. LJEO showed certain removal effects against mature biofilms of *L. monocytogenes*, *E. coli*, and *S. paratyphi* B, but showed limited activity against mature *S. aureus* biofilms. This finding suggests that inhibition of planktonic cells does not necessarily predict the ability to remove established biofilms. Cells within mature biofilms are protected by extracellular polymeric substances, which can restrict the penetration of antimicrobial compounds and increase bacterial tolerance to environmental stress. Previous studies have emphasized that biofilms in food processing environments serve as important reservoirs for persistent contamination and cross-contamination, and that the EPS matrix can enhance resistance to cleaning agents, disinfectants, and antimicrobial treatments [37,38]. Therefore, the strong inhibitory effect of LJEO against planktonic *S. aureus* but weak removal effect against mature *S. aureus* biofilms may be related to the compact biofilm architecture and stronger extracellular matrix barrier of this strain. These results further suggest that both planktonic inhibition and mature biofilm removal should be considered when evaluating the application value of essential oils for food microbial safety.

The bacterial growth curve and SEM results provided additional evidence for the antibacterial effect of LJEO. LJEO at MIC maintained low OD600 values during the tested period, while sub-inhibitory concentrations delayed bacterial growth to different degrees, indicating a concentration-dependent inhibitory effect. SEM observation showed that LJEO-treated bacterial cells exhibited shrinkage, depression, collapse, surface disruption, and morphological distortion, suggesting that LJEO may impair cell surface structures and affect cell envelope integrity. Similar cellular damage has been reported in studies of other plant essential oils. For example, Fingered Citron essential oil was shown to induce morphological changes in *L. monocytogenes*, lemon essential oil nanoemulsions affected the membrane integrity, membrane potential, and efflux function of *E. coli*, and *Cinnamomum camphora* essential oil was reported to inhibit bacterial growth through membrane damage, zeta potential alteration, and oxidative stress [4,39,40]. These observations are consistent with the growth curve and SEM results in the present study and suggest that LJEO may inhibit bacterial growth through combined effects on cell surface structures and cellular homeostasis.

From the perspective of food application, LJEO may have potential as a natural antimicrobial and preservative candidate. *L. japonica* is a medicinal and edible plant resource, and its extracts have been reported to possess antibacterial, antioxidant, and preservation-related activities [30]. The present study further suggests that LJEO can inhibit several foodborne pathogens and remove some mature bacterial biofilms to different degrees. However, the application of essential oils in real food systems still faces several challenges, including high volatility, strong hydrophobicity, limited stability under heat, oxygen, and light exposure, pronounced aroma that may negatively affect the sensory quality of foods and reduce consumer acceptance, and possible adsorption or encapsulation of active components by food matrices [41]. Therefore, future work should evaluate the antibacterial performance, sensory impact, toxicological safety regulatory approval requirements, industrial scalability, and storage stability of LJEO in real food systems. Formulation strategies such as nanoemulsions, edible films, active packaging, or combined preservation systems may also help improve its dispersion, stability, and controlled release in food environments.

Overall, this study showed that the nine TCM herb essential oils differed markedly in antibacterial activity and volatile composition. LJEO exhibited relatively favorable antibacterial activity and a distinct volatile profile, and its activity may be associated with the combined contribution of linalool, borneol, isoborneol, 1,8-cineole, p-cymene, and other oxygenated terpenes. The enrichment of eugenol, cinnamaldehyde, borneol, and 1,8-cineole in SAEO and SBEO may also contribute to their antibacterial effects. Although LJEO showed limited removal activity against mature *S. aureus* biofilms and a relatively higher MIC against *E. coli*, it still displayed promising inhibitory effects against planktonic cells of multiple foodborne pathogens and certain mature biofilms. Nevertheless, because the biofilm experiments were performed using a single concentration, the relative biofilm removal performance observed in this study should not be interpreted as a complete characterization of the antibiofilm potency of LJEO or the other essential oils. Further studies integrating dose–response evaluation, single-compound validation, synergistic interaction analysis, mechanistic assays, and real food system evaluation will be necessary to clarify the practical value of LJEO and other active TCM herb essential oils as natural antimicrobial and preservative candidates for food safety applications.

## 5. Conclusions

This study systematically compared the antibacterial activity, mature biofilm removal ability, and volatile composition of nine TCM herb essential oils. The results showed clear differences in antibacterial performance and chemical profiles among the tested oils. Among them, *Lonicera japonica*, *Sinomenium acutum*, and *Scutellaria baicalensis* essential oils exhibited relatively broad antibacterial activity under the present experimental conditions. In particular, LJEO showed low MIC values against *S. aureus* and *L. monocytogenes* and displayed certain removal effects against mature biofilms of *L. monocytogenes*, *E. coli*, and *S. paratyphi* B, although its effect on mature *S. aureus* biofilms was limited. These results indicate that planktonic cell inhibition does not necessarily correspond to mature biofilm removal ability. GC–MS analysis identified 156 volatile compounds across the nine essential oils. LJEO showed a distinct volatile profile, with a relatively high number of unique compounds and abundant linalool, p-cymene, borneol, cineoles, and limonene. Correlation analysis further suggested that eugenol, borneol, isoborneol, cinnamaldehyde, carveol, γ-terpineol, α-cadinol, and nerolidol may be associated with antibacterial activity. Growth curve and SEM analyses indicated that LJEO inhibited bacterial proliferation and induced cell surface shrinkage, depression, and structural damage, suggesting that its antibacterial effect may be partly related to impairment of bacterial cell surface structures. Overall, this study identified selected TCM herb essential oils with potential food antimicrobial applications, particularly LJEO, which may serve as a natural antimicrobial candidate for foodborne pathogen control and food preservation. Further studies involving single-compound validation, synergistic interaction analysis, mechanistic investigation, and real food system evaluation are needed to clarify its safety, stability, and practical application value.

## Figures and Tables

**Table 1:** Qualitative identification of volatile compounds in nine TCM herb essential oils by GC–MS.

RT	RI^a^	RI^b^	CAS	Molecular Formula	Class I	Name
4.399	909	925	508-32-7	C_10_H_16_	Monoterpenes	Tricyclene
4.659	921	929	2867-05-2	C_10_H_16_	Monoterpenes	alpha-Thujene
4.931	933	952	79-92-5	C_10_H_16_	Monoterpenes	Camphene
5.542	960	/	18172-67-3	C_10_H_16_	Monoterpenes	(-)-beta-Pinene
5.565	961	974	3387-41-5	C_10_H_16_	Monoterpenes	Sabinene
5.797	971	979	127-91-3	C_10_H_16_	Monoterpenes	beta-Pinene
6.195	989	1005	99-83-2	C_10_H_16_	Monoterpenes	alpha-Phellandrene
6.351	996	1011	13466-78-9	C_10_H_16_	Monoterpenes	3-Carene
6.495	1001	1016	470-67-7	C_10_H_18_O	Ethers	1,4-Cineole
6.549	1003	1017	99-86-5	C_10_H_16_	Monoterpenes	alpha-Terpinene
6.73	1009	1025	99-87-6	C_10_H_14_	Others	p-Cymene
6.866	1013	1030	138-86-3	C_10_H_16_	Monoterpenes	Limonene, (-)-
6.99	1017	1032	470-82-6	C_10_H_18_O	Alcohols	1,8-Cineole
7.366	1029	1047	7416-35-5	C_10_H_18_O	Ethers	2,2-Dimethyl-5-(1-methylpropenyl)tetrahydrofuran
7.725	1040	1060	99-85-4	C_10_H_16_	Monoterpenes	gamma-Terpinene
8.572	1067	1084	27644-04-8	C_10_H_18_O	Alcohols	3,3,6-Trimethyl-1,5-heptadien-4-ol
8.674	1070	1088	586-62-9	C_10_H_16_	Monoterpenes	Terpinolene
9.03	1081	1095	1686-14-2	C_10_H_16_O	Ethers	alpha-Pinene oxide
9.086	1083	1099	78-70-6	C_10_H_18_O	Alcohols	Linalool
9.297	1090	1102	3886-78-0	C_10_H_16_O	Alcohols	Filifolone
9.358	1092	1103	546-80-5	C_10_H_16_O	Ketones	Thujone
9.533	1097	1113	1632-73-1	C_10_H_18_O	Alcohols	Fenchol
9.717	1103	1114	471-15-8	C_10_H_16_O	Ketones	beta-Thujone
9.856	1106	/	42393-93-1	C_10_H_16_O_2_	Ketones	Cis-bicyclo[4.4.0]decan-1-ol-3-one
9.913	1108	1122	29803-82-5	C_10_H_18_O	Alcohols	2-Cyclohexen-1-ol, 1-methyl-4-(1-methylethyl)-, (1R,4R)-rel-
10.011	1110	1123	473-06-3	C_10_H_14_O	Ketones	Chrysanthenone
10.267	1117	1137	586-82-3	C_10_H_18_O	Alcohols	p-Menth-3-en-1-ol
10.569	1125	1140	29803-81-4	C_10_H_18_O	Alcohols	cis-p-Menth-2-en-1-ol
10.648	1127	/	1845-30-3	C_10_H_16_O	Alcohols	Verbenol, (S)-cis-
10.72	1129	1142	464-48-2	C_10_H_16_O	Alcohols	Camphor, (-)-
10.796	1131	/	206115-88-0	C_10_H_16_O	Alcohols	(3E,5E)-2,6-Dimethylocta-3,5,7-trien-2-ol
11.019	1137	/	14073-97-3	C_10_H_18_O	Ketones	Menthone
11.104	1139	1157	124-76-5	C_10_H_18_O	Alcohols	Isoborneol
11.41	1147	1142	38043-83-3	C_10_H_16_O	Alcohols	trans-Chrysanthenol
11.455	1148	1167	507-70-0	C_10_H_18_O	Alcohols	Borneol
11.871	1159	1070	17699-16-0	C_10_H_18_O	Alcohols	4-Thujanol
11.976	1162	1177	562-74-3	C_10_H_18_O	Alcohols	Terpinen-4-ol
12.169	1167	1183	1197-01-9	C_10_H_14_O	Alcohols	p-Cymen-8-ol
12.391	1173	1189	98-55-5	C_10_H_18_O	Alcohols	alpha-Terpineol
12.637	1179	1197	586-81-2	C_10_H_18_O	Alcohols	gamma-Terpineol
12.654	1180	1196	140-67-0	C_10_H_12_O	Ethers	Estragole
12.67	1180	/	NIST#: 131117	C_19_H_34_	Others	(4Z,6Z,9Z)-4,6,9-Nonadecatriene
12.858	1185	/	43142-43-4	C_9_H_12_O	Alcohols	3,5-Nonadien-7-yn-2-ol, (E,E)-
13.155	1193	1195	16721-38-3	C_10_H_18_O	Alcohols	cis-Piperitol
13.601	1204	1217	1197-07-5	C_10_H_16_O	Alcohols	Carveol, trans-(-)-
13.631	1205	/	54274-41-8	C_10_H_14_O	Alcohols	3-Cyclohexen-1-ol, 5-methylene-6-(1-methylethenyl)-
14.041	1215	1235	1076-56-8	C_11_H_16_O	Ethers	Thymyl methyl ether
14.183	1219	1219	99-48-9	C_10_H_16_O	Alcohols	Carveol
14.277	1221	/	529-00-0	C_10_H_16_O	Ketones	Cyclohexanone, 5-methyl-2-(1-methylethenyl)-
14.899	1236	/	NIST#: 99256	C_12_H_18_O	Others	4-Isopropyl-trans-bicyclo[4.3.0]-2-nonen-8-one, (4R,S)-
15.054	1240	1251	123-11-5	C_8_H_8_O_2_	Aldehydes	4-Methoxybenzaldehyde
15.554	1252	1270	14371-10-9	C_9_H_8_O	Aldehydes	Cinnamaldehyde
16.019	1264	/	NIST#: 184988	C_12_H_16_O	Alcohols	Tetracyclo[4.4.1.1(7,10).0(2,5)]dodec-3-en-11-ol
16.089	1265	1285	76-49-3	C_12_H_20_O_2_	Esters	Bornyl acetate, (-)-
16.217	1268	1284	4180-23-8	C_10_H_12_O	Ethers	Anethole
16.241	1269	1254	25679-28-1	C_10_H_12_O	Ethers	cis-Anethole
16.662	1279	1297	1686-15-3	C_12_H_18_O_2_	Esters	Pinocarvyl acetate, trans-(+)-
16.72	1281	/	NIST#: 193387	C_10_H_14_O	Alcohols	Tricyclo[4.4.0.0(2,8)]dec-3-en-5-ol
16.996	1287	/	33240-56-1	C_7_H_15_Cl	Others	Hexane, 1-chloro-5-methyl-
17.09	1290	/	80255-20-5	C_9_H_13_NO_2_	Others	Cyclohexene, 1-(2-nitro-2-propenyl)-
17.229	1293	1294	NIST#: 195921	C_14_H_22_O	Ethers	1-Oxaspiro[2.5]octane, 5,5-dimethyl-4-(3-methyl-1,3-butadienyl)-
17.456	1299	/	943-93-1	C_12_H_18_O	Ethers	1,2-Epoxy-5,9-cyclododecadiene
17.599	1302	/	NIST#: 99285	C_12_H_18_O_2_	Aldehydes	(S,S,S,S)-1,1′-Bicyclopentyl-2,2′-dicarboxaldehyde
17.646	1303	1330	22539-72-6	C_10_H_16_O	Alcohols	p-Mentha-1,4-dien-7-ol
17.782	1307	/	13837-66-6	C_10_H_20_	Others	Cyclohexane, 1-methyl-3-(1-methylethyl)-, cis-
17.923	1310	/	NIST#: 374169	C_10_H_18_O_2_	Alcohols	1-Methyl-4-(propan-2-yl)cyclohex-2-ene-1,4-diol
18.576	1326	/	29205-99-0	C_11_H_14_O_2_	Acids	2-Methyl-2-(2-methylphenyl)propanoic acid
18.756	1330	1353	5989-08-2	C_15_H_24_	Sesquiterpenes	alpha-Longipinene
19.121	1339	1357	97-53-0	C_10_H_12_O_2_	Phenols	Eugenol
19.286	1343	/	21632-06-4	C_14_H_24_O	Aldehydes	alpha,2,6,6-Tetramethyl-1-cyclohexene-1-butanal
19.551	1350	1372	14912-44-8	C_15_H_24_	Sesquiterpenes	Ylangene
19.615	1351	1375	501-19-9	C_10_H_12_O_2_	Phenols	Chavibetol
20.026	1361	1373	121-98-2	C_9_H_10_O_3_	Esters	Methyl p-anisate
20.148	1364	/	NIST#: 185672 ID	C_12_H_18_O	Ethers	1-Methyl-6-(3-methylbuta-1,3-dienyl)-7-oxabicyclo[4.1.0]heptane
20.25	1367	1390	1135-66-6	C_15_H_24_	Sesquiterpenes	Isolongifolene
20.396	1370	1384	122-84-9	C_10_H_12_O_2_	Ketones	1-(p-Methoxyphenyl)-2-propanone
20.981	1384	1403	41432-70-6	C_15_H_24_	Sesquiterpenes	beta-Longipinene
21.291	1392	1389	65354-33-8	C_15_H_24_	Sesquiterpenes	2-epi-alpha-Funebrene
21.542	1398	1419	87-44-5	C_15_H_24_	Sesquiterpenes	(-)-Caryophyllene
21.777	1404	/	21966-93-8	C_15_H_26_O	Alcohols	(1S,3aS,4S,5S,7aR,8R)-5-Isopropyl-1,7a-dimethyloctahydro-1H-1,4-methanoinden-8-ol
22.026	1410	/	NIST#: 188358	C_12_H_20_O_2_	Ethers	6-Isopropenyl-3-methoxymethoxy-3-methyl-cyclohexene
22.035	1410	1429	470-40-6	C_15_H_24_	Sesquiterpenes	Thujopsene
22.262	1416	1435	13474-59-4	C_15_H_24_	Sesquiterpenes	trans-alpha-Bergamotene
22.347	1418	1440	489-39-4	C_15_H_24_	Sesquiterpenes	Aromandendrene
22.821	1430	/	5561-92-2	C_10_H_12_O_2_	Ketones	1-(2-Methoxyphenyl)propan-1-one
22.886	1431	1454	6753-98-6	C_15_H_24_	Sesquiterpenes	Humulene
23.002	1434	/	242794-76-9	C_15_H_24_	Sesquiterpenes	4,8,8-Trimethyl-2-methylene-4-vinylbicyclo(5.2.0)nonane
23.148	1438	1444	28973-97-9	C_15_H_24_	Sesquiterpenes	beta-Farnesene, (6Z)-
23.248	1440	1435	20071-49-2	C_15_H_24_	Sesquiterpenes	gamma-Maaliene
23.493	1447	1443	36577-33-0	C_15_H_24_	Sesquiterpenes	6,9-Guaiadiene
23.74	1453	1476	18431-82-8	C_15_H_24_	Sesquiterpenes	beta-Chamigrene
24.104	1462	1481	23986-74-5	C_15_H_24_	Sesquiterpenes	Germacrene D
24.145	1463	/	NIST#: 430950	C_12_H_18_O_3_	Ethers	p-(Diethoxymethyl)anisole
24.221	1465	1474	37079-64-4	C_13_H_20_O_2_	Ketones	7,8-Epoxy-alpha-ionone
24.293	1466	1486	17066-67-0	C_15_H_24_	Sesquiterpenes	beta-Selinene
24.3	1467	1473	22567-17-5	C_15_H_24_	Sesquiterpenes	gamma-Gurjunene
24.401	1469	1480	451-55-8	C_15_H_24_	Sesquiterpenes	1-Methyl-4-(6-methylhept-5-en-2-yl)cyclohexa-1,3-diene
24.756	1478	1492	93-16-3	C_11_H_14_O_2_	Ethers	Methylisoeugenol
24.78	1479	/	31983-22-9	C_15_H_24_	Sesquiterpenes	alpha-Muurolene
24.877	1481	1479	82462-31-5	C_15_H_22_	Sesquiterpenes	Eudesma-2,4,11-triene
24.981	1484	1502	107-50-6	C_14_H_42_O_7_Si_7_	Ethers	Tetradecamethylcycloheptasiloxane
25.022	1485	/	NIST#: 295457	C_15_H_13_Cl_2_NO_2_S	Others	Quinoline, 1,2,3,4-tetrahydro-1-(2,5-dichlorophenylsulfonyl)-
25.174	1488	/	80923-88-2	C_15_H_24_	Sesquiterpenes	alpha-Himachalene
25.228	1490	1505	16982-00-6	C_15_H_22_	Others	Cuparene
25.297	1491	1495	24703-35-3	C_15_H_24_	Sesquiterpenes	Bicyclogermacrene
25.337	1492	1492	4630-07-3	C_15_H_24_	Sesquiterpenes	Valencene
25.458	1496	1452	3293-47-8	C_13_H_24_O	Alcohols	Dihydro-beta-ionol
25.513	1497	/	NIST#: 430806	C_13_H_18_O_2_	Ethers	(3,3-Diethoxy-1-propen-1-yl)benzene
25.609	1499	1493	38230-60-3	C_15_H_26_O	Alcohols	epi-Cubebol
25.646	1500	/	NIST#: 189126	C_14_H_22_O_2_	Ketones	1-[3,3-Dimethyl-2-(3-methyl-buta-1,3-dienyl)-cyclopentyl]-2-hydroxy-ethanone
25.729	1502	1524	483-76-1	C_15_H_24_	Sesquiterpenes	delta-Cadinene
25.905	1507	1514	28976-67-2	C_15_H_24_	Sesquiterpenes	(-)-beta-Curcumene
26.792	1529	/	74744-55-1	C_17_H_30_	Others	3-Heptadecen-5-yne, (Z)-
27.32	1542	1564	40716-66-3	C_15_H_26_O	Alcohols	Nerolidol
27.736	1552	1576	6750-60-3	C_15_H_24_O	Alcohols	Spathulenol
27.92	1557	1581	1139-30-6	C_15_H_24_O	Ethers	Caryophyllene oxide
28.316	1567	/	NIST#: 153997	C_13_H_18_O	Ketones	Tricyclo[6.3.0.0(1,5)]undec-2-en-4-one, 5,9-dimethyl-
28.716	1577	1598	77-53-2	C_15_H_26_O	Alcohols	Cedrol
28.885	1581	/	87096-70-6	C_10_H_18_O_3_	Others	1,2-cis-1,5-trans-2,5-dihydroxy-4-methyl-1-(1-hydroxy-1-isopropyl)cyclohex-3-ene
29.225	1590	1606	19888-34-7	C_15_H_24_O	Ethers	HumuleneepoxideII
29.247	1590	/	NIST#: 191493	C_14_H_18_O	Ketones	11-Isopropylidenetricyclo[4.3.1.1(2,5)]undec-3-en-10-one
29.757	1603	1649	28400-11-5	C_15_H_26_O	Alcohols	beta-Acorenol
30.469	1618	/	5951-61-1	C_15_H_24_	Sesquiterpenes	1-Isopropyl-4,7-dimethyl-1,2,4a,5,8,8a-hexahydronaphthalene
30.503	1618	1631	19890-84-7	C_15_H_24_O	Aldehydes	Longifolenaldehyde
30.677	1622	/	54594-42-2	C_14_H_24_O	Ketones	2(1H)-Naphthalenone, octahydro-4a-methyl-7-(1-methylethyl)-, (4aalpha,7beta,8abeta)-
30.974	1628	1650	19888-00-7	C_15_H_24_O	Alcohols	Humulenol
31.065	1630	1653	481-34-5	C_15_H_26_O	Alcohols	alpha-Cadinol
31.167	1632	1636	118173-08-3	C_15_H_26_O	Alcohols	Selin-6-en-4alpha-ol
32.19	1654	/	NIST#: 71702	C_19_H_36_O_5_Si_3_	Esters	3,4-Dihydroxymandelic acid, ethyl ester, tri-TMS
32.217	1655	1704	556-68-3	C_16_H_48_O_8_Si_8_	Ethers	Hexadecamethylcyclooctasiloxane
32.414	1659	1684	78259-41-3	C_14_H_18_O	Ethers	1-(3-Methyl-2-butenoxy)-4-(1-propenyl)benzene
32.465	1660	1678	2883-98-9	C_12_H_16_O_3_	Ethers	Asarone
32.971	1671	/	19903-72-1	C_15_H_24_O	Alcohols	Santalol, cis,alpha-
34.153	1696	1688	18319-35-2	C_15_H_24_O	Alcohols	Cedren-13-ol, 8-
35.571	1726	1777	135118-51-3	C_15_H_24_O	Alcohols	alpha-Muurolene-14-ol
36.778	1751	/	NIST#: 190222	C_15_H_22_O	Ethers	4,6,6-Trimethyl-2-(3-methylbuta-1,3-dienyl)-3-oxatricyclo[5.1.0.0(2,4)]octane
36.928	1755	1770	76252-27-2	C_13_H_18_O_3_	Ethers	(E)-1-(2,4,5-Trimethoxyphenyl)but-1-ene
37.225	1761	1778	65018-15-7	C_15_H_24_O	Alcohols	(-)-alpha-Costol
37.725	1771	1756	562-23-2	C_15_H_22_O	Ketones	Eremophilone
37.887	1775	1792	132342-55-3	C_16_H_24_O_2_	Esters	Methyl isocostate
39.787	1815	1893	556-71-8	C_18_H_54_O_9_Si_9_	Ethers	Octadecamethylcyclononasiloxane
40.28	1825	/	NIST#: 196922	C_16_H_32_O_2_Si	Ketones	5-(2-Hydroxy-1-methylethyl)-2-methylcyclohexanone, TBDMS derivative
46.01	1948	/	77026-88-1	C_10_H_16_O_2_	Others	(3R,6R)-3-Hydroperoxy-3-methyl-6-(prop-1-en-2-yl)cyclohex-1-ene
46.817	1965	2035	18772-36-6	C_20_H_60_O_10_Si_10_	Ethers	Eicosamethylcyclodecasiloxane
46.87	1966	2453	212516-36-4	C_18_H_20_O_4_	Ethers	rel-(+)-(2R,4R,5S)-2,4-Bis(4-methoxyphenyl)-5-methyl-1,3-dioxolane
46.904	1967	/	19095-24-0	C_16_H_50_O_7_Si_8_	Ethers	Octasiloxane, 1,1,3,3,5,5,7,7,9,9,11,11,13,13,15,15-hexadecamethyl-
48.789	2008	/	205692-58-6	C_17_H_16_O_3_	Ethers	Cambridge id 5364929
52.38	2088	2503	844679-34-1	C_20_H_24_O_3_	Ethers	2,4-Bis(4-methoxyphenyl)-3,5-dimethyltetrahydrofuran
53.663	/	/	382-67-2	C_22_H_29_FO_4_	Ketones	Desoximetasone
56.043	/	/	124-94-7	C_21_H_27_FO_6_	Ketones	Triamcinolone
59.867	/	/	19095-23-9	C_14_H_44_O_6_Si_7_	Ethers	AC1LB8OT
60.55	/	/	NIST#: 124602	C_33_H_54_O_3_	Esters	Cholest-22-ene-21-ol, 3,5-dehydro-6-methoxy-, pivalate
60.776	/	/	24560-98-3	C_18_H_34_O_3_	Acids	9,10-Epoxystearic acid, cis-
61.203	/	2561	177717-46-3	C_21_H_40_O_5_	Esters	Decanoic acid, ester with 1,2,3-propanetriol octanoate
61.389	/	2376	1429-66-9	C_19_H_36_O_5_	Esters	1,3-Dioctanoyl glycerol
61.645	/	2381	60514-48-9	C_19_H_36_O_5_	Acids	1,2-Dioctanoyl-sn-glycerol

RI^a^: Retention index calculated on HP-5ms capillary column. RI^b^: The retention index was calculated using the retention times (RT) of n-alkanes and the compounds of interest. “/”: This compound does not currently have a CAS number; the number listed in the table corresponds to the identifier from the NIST spectral library.

**Table 2:** Relative contents of volatile compounds in nine TCM herb essential oils.

RT	Name	SAEO	LJEO	SBEO	PCEO	PLEO	PFEO	GUEO	AAEO	LBEO
4.399	Tricyclene	0.15	0.28	-	1.43	-	0.43	-	-	0.62
4.659	alpha-Thujene	19.96	4.26	1.83	0.18	0.97	25.83	1.17	4.12	41.38
4.931	Camphene	1.38	2.93	0.18	-	0.14	3.65	0.15	0.67	4.93
5.542	(-)-beta-Pinene	0.42	-	-	-	-	-	-	0.15	-
5.565	Sabinene	-	-	-	-	-	-	-	0.14	-
5.797	beta-Pinene	-	0.27	-	-	-	-	-	-	-
6.195	alpha-Phellandrene	-	0.15	-	-	-	-	-	-	-
6.351	3-Carene	-	0.36	-	-	-	0.51	-	-	-
6.495	1,4-Cineole	-	6.10	0.08	0.05	-	2.21	-	0.23	1.54
6.549	alpha-Terpinene	-	-	-	-	-	-	-	0.30	0.47
6.73	p-Cymene	1.58	6.72	0.73	0.15	0.14	3.77	0.12	0.93	2.66
6.866	Limonene, (-)-	6.06	5.88	2.18	0.36	0.12	6.00	0.39	1.18	5.05
6.99	1,8-Cineole	14.63	5.90	6.62	0.36	0.21	6.83	0.30	3.38	8.07
7.366	2,2-Dimethyl-5-(1-methylpropenyl)tetrahydrofuran	-	0.09	-	-	-	-	-	-	-
7.725	gamma-Terpinene	0.33	1.93	0.11	0.02	-	1.21	-	0.52	1.10
8.572	3,3,6-Trimethyl-1,5-heptadien-4-ol	-	-	-	-	-	-	-	0.04	-
8.674	Terpinolene	0.27	0.01	0.21	0.17	0.10	4.76	0.14	0.97	1.96
9.03	alpha-Pinene oxide	0.80	-	-	-	-	-	-	-	-
9.086	Linalool	-	10.30	-	-	-	3.02	-	-	-
9.297	Filifolone	0.41	-	-	-	-	-	-	-	-
9.358	Thujone	-	-	-	-	-	-	-	2.23	-
9.533	Fenchol	-	1.11	-	-	-	-	-	-	-
9.717	beta-Thujone	-	-	-	-	-	-	-	0.37	-
9.856	Cis-bicyclo[4.4.0]decan-1-ol-3-one	-	0.09	-	-	-	-	-	-	-
9.913	2-Cyclohexen-1-ol, 1-methyl-4-(1-methylethyl)-, (1R,4R)-rel-	-	-	-	-	-	-	-	0.26	-
10.011	Chrysanthenone	-	-	-	-	-	-	-	0.63	-
10.267	p-Menth-3-en-1-ol	-	0.45	-	-	-	-	-	-	-
10.569	cis-p-Menth-2-en-1-ol	-	-	-	-	-	-	-	0.40	-
10.648	Verbenol, (S)-cis-	0.66	1.41	-	-	-	-	-	-	-
10.72	Camphor, (-)-	-	-	-	-	-	-	-	0.98	-
10.796	(3E,5E)-2,6-Dimethylocta-3,5,7-trien-2-ol	-	0.17	-	-	-	-	-	-	-
11.019	Menthone	-	4.71	-	-	-	0.23	-	-	-
11.104	Isoborneol	4.18	4.43	2.09	-	-	-	-	-	-
11.41	trans-Chrysanthenol	-	-	-	-	-	-	-	0.15	-
11.455	Borneol	5.57	6.44	2.74	-	-	-	-	1.06	-
11.871	4-Thujanol	-	0.14	-	-	-	-	-	-	-
11.976	Terpinen-4-ol	-	-	-	-	-	-	-	0.75	-
12.169	p-Cymen-8-ol	-	1.17	-	-	-	-	-	-	-
12.391	alpha-Terpineol	-	1.54	-	-	-	-	-	0.38	-
12.637	gamma-Terpineol	-	0.43	-	-	-	-	-	-	-
12.654	Estragole	0.17	-	0.06	0.10	-	-	0.68	-	-
12.67	(4Z,6Z,9Z)-4,6,9-Nonadecatriene	-	-	-	-	-	-	-	0.04	-
12.858	3,5-Nonadien-7-yn-2-ol, (E,E)-	-	0.42	-	-	-	-	-	-	-
13.155	cis-Piperitol	-	-	-	-	-	-	-	0.14	-
13.601	Carveol, trans-(-)-	-	-	-	-	-	-	-	0.15	-
13.631	3-Cyclohexen-1-ol, 5-methylene-6-(1-methylethenyl)-	-	0.06	-	-	-	-	-	-	-
14.041	Thymyl methyl ether	-	-	-	-	-	-	-	-	0.14
14.183	Carveol	-	0.27	-	-	-	-	-	-	-
14.277	Cyclohexanone, 5-methyl-2-(1-methylethenyl)-	-	1.48	-	-	0.16	-	-	-	-
14.899	4-Isopropyl-trans-bicyclo[4.3.0]-2-nonen-8-one, (4R,S)-	-	0.16	-	0.11	-	0.37	-	-	-
15.054	4-Methoxybenzaldehyde	0.33	-	6.09	-	-	-	1.06	-	-
15.554	Cinnamaldehyde	9.49	-	3.97	-	-	-	-	-	-
16.019	Tetracyclo[4.4.1.1(7,10).0(2,5)]dodec-3-en-11-ol	-	0.37	-	-	-	-	-	-	-
16.089	Bornyl acetate, (-)-	-	-	-	-	-	-	-	-	0.64
16.217	Anethole	16.48	-	8.64	6.75	-	0.83	4.25	-	-
16.241	cis-Anethole	-	-	-	-	-	-	-	0.31	-
16.662	Pinocarvyl acetate, trans-(+)-	1.13	-	-	-	-	-	5.20	-	-
16.72	Tricyclo[4.4.0.0(2,8)]dec-3-en-5-ol	-	0.25	-	-	-	-	-	-	-
16.996	Hexane, 1-chloro-5-methyl-	0.16	-	-	-	-	-	-	-	-
17.09	Cyclohexene, 1-(2-nitro-2-propenyl)-	-	0.39	-	-	-	-	-	-	-
17.229	1-Oxaspiro[2.5]octane, 5,5-dimethyl-4-(3-methyl-1,3-butadienyl)-	-	0.20	-	-	-	-	-	-	-
17.456	1,2-Epoxy-5,9-cyclododecadiene	-	0.86	-	-	-	-	-	-	-
17.599	(S,S,S,S)-1,1′-Bicyclopentyl-2,2′-dicarboxaldehyde	-	0.18	-	-	-	-	-	-	-
17.646	p-Mentha-1,4-dien-7-ol	0.25	-	-	-	-	-	-	-	-
17.782	Cyclohexane, 1-methyl-3-(1-methylethyl)-, cis-	-	0.99	-	-	-	-	-	-	-
17.923	1-Methyl-4-(propan-2-yl)cyclohex-2-ene-1,4-diol	-	0.32	-	-	-	-	-	-	-
18.576	2-Methyl-2-(2-methylphenyl)propanoic acid	-	2.38	-	-	-	-	-	-	-
18.756	alpha-Longipinene	-	0.40	-	-	-	-	-	-	-
19.121	Eugenol	12.27	-	10.98	-	-	-	-	0.15	-
19.286	alpha,2,6,6-Tetramethyl-1-cyclohexene-1-butanal	-	0.18	-	-	-	-	-	-	-
19.551	Ylangene	-	-	-	-	-	-	-	-	10.12
19.615	Chavibetol	-	0.32	25.25	-	-	-	-	-	-
20.026	Methyl p-anisate	-	-	0.28	-	-	-	-	-	-
20.148	1-Methyl-6-(3-methylbuta-1,3-dienyl)-7-oxabicyclo[4.1.0]heptane	-	0.57	-	-	-	-	-	-	-
20.25	Isolongifolene	-	0.34	-	-	-	-	-	-	-
20.396	1-(p-Methoxyphenyl)-2-propanone	-	0.16	1.60	-	-	-	-	-	-
20.981	beta-Longipinene	-	3.31	-	-	-	-	-	-	-
21.291	2-epi-alpha-Funebrene	-	-	-	-	-	0.18	-	-	-
21.542	(-)-Caryophyllene	1.34	0.54	5.01	-	-	4.01	-	2.23	-
21.777	(1S,3aS,4S,5S,7aR,8R)-5-Isopropyl-1,7a-dimethyloctahydro-1H-1,4-methanoinden-8-ol	-	0.13	-	-	-	-	-	-	-
22.026	6-Isopropenyl-3-methoxymethoxy-3-methyl-cyclohexene	-	1.56	-	-	-	-	-	-	-
22.035	Thujopsene	-	-	-	-	-	0.14	-	-	-
22.262	trans-alpha-Bergamotene	-	-	0.66	-	-	-	-	-	-
22.347	Aromandendrene	-	0.29	0.06	-	-	-	-	-	-
22.821	1-(2-Methoxyphenyl)propan-1-one	-	-	0.16	-	-	-	-	-	-
22.886	Humulene	0.36	0.32	-	-	-	0.22	-	0.24	-
23.002	4,8,8-Trimethyl-2-methylene-4-vinylbicyclo(5.2.0)nonane	-	-	1.86	-	-	-	-	-	-
23.148	beta-Farnesene, (6Z)-	-	-	-	-	-	0.62	-	-	-
23.248	gamma-Maaliene	-	0.39	-	-	-	-	-	-	-
23.493	6,9-Guaiadiene	-	0.07	-	-	-	-	-	-	-
23.74	beta-Chamigrene	-	-	-	-	-	-	-	-	1.71
24.104	Germacrene D	-	-	-	-	-	-	-	1.05	-
24.145	p-(Diethoxymethyl)anisole	-	-	1.98	-	-	-	-	-	-
24.221	7,8-Epoxy-alpha-ionone	-	0.93	-	-	-	-	-	-	-
24.293	beta-Selinene	-	-	-	-	-	-	-	0.40	-
24.3	gamma-Gurjunene	-	1.16	-	-	-	-	-	-	-
24.401	1-Methyl-4-(6-methylhept-5-en-2-yl)cyclohexa-1,3-diene	-	-	-	-	-	-	-	-	3.62
24.756	Methylisoeugenol	-	-	1.06	-	-	-	-	-	-
24.78	alpha-Muurolene	-	-	-	-	-	0.14	-	-	-
24.877	Eudesma-2,4,11-triene	-	-	-	-	-	-	-	-	1.28
24.981	Tetradecamethylcycloheptasiloxane	-	-	-	-	-	-	-	-	0.31
25.022	Quinoline, 1,2,3,4-tetrahydro-1-(2,5-dichlorophenylsulfonyl)-	-	-	-	-	-	0.23	-	-	-
25.174	alpha-Himachalene	-	-	0.86	-	-	-	-	-	-
25.228	Cuparene	-	-	-	-	-	-	-	0.06	-
25.297	Bicyclogermacrene	-	-	-	-	-	0.14	-	0.09	-
25.337	Valencene	-	-	0.26	-	-	-	-	-	-
25.458	Dihydro-beta-ionol	-	2.99	-	-	-	-	-	-	-
25.513	(3,3-Diethoxy-1-propen-1-yl)benzene	0.62	-	1.08	-	-	-	-	-	-
25.609	epi-Cubebol	-	-	-	-	-	-	-	-	0.33
25.646	1-[3,3-Dimethyl-2-(3-methyl-buta-1,3-dienyl)-cyclopentyl]-2-hydroxy-ethanone	-	0.24	-	-	-	-	-	-	-
25.729	delta-Cadinene	-	-	0.96	-	-	0.27	-	0.13	-
25.905	(-)-beta-Curcumene	-	-	-	-	-	-	-	-	0.64
26.792	3-Heptadecen-5-yne, (Z)-	-	-	0.10	-	-	-	-	-	-
27.32	Nerolidol	-	-	0.76	-	-	-	-	-	-
27.736	Spathulenol	-	-	-	-	-	0.20	-	0.05	-
27.92	Caryophyllene oxide	0.14	0.17	1.31	-	-	0.90	-	0.40	-
28.316	Tricyclo[6.3.0.0(1,5)]undec-2-en-4-one, 5,9-dimethyl-	-	-	0.36	-	-	-	-	-	-
28.716	Cedrol	-	-	-	-	-	0.11	-	-	-
28.885	1,2-cis-1,5-trans-2,5-dihydroxy-4-methyl-1-(1-hydroxy-1-isopropyl)cyclohex-3-ene	-	0.26	-	-	-	-	-	-	-
29.225	HumuleneepoxideII	-	1.05	-	-	-	-	-	-	-
29.247	11-Isopropylidenetricyclo[4.3.1.1(2,5)]undec-3-en-10-one	-	-	0.13	-	-	-	-	-	-
29.757	beta-Acorenol	-	-	-	-	-	0.22	-	-	-
30.469	1-Isopropyl-4,7-dimethyl-1,2,4a,5,8,8a-hexahydronaphthalene	-	-	0.57	-	-	-	-	-	-
30.503	Longifolenaldehyde	-	2.26	-	-	-	-	-	-	-
30.677	2(1H)-Naphthalenone, octahydro-4a-methyl-7-(1-methylethyl)-, (4aalpha,7beta,8abeta)-	-	0.14	-	-	-	-	-	-	-
30.974	Humulenol	-	0.14	-	-	-	-	-	-	-
31.065	alpha-Cadinol	-	-	0.69	-	-	-	-	-	-
31.167	Selin-6-en-4alpha-ol	-	-	-	-	-	-	-	1.00	-
32.19	3,4-Dihydroxymandelic acid, ethyl ester, tri-TMS	-	-	-	-	-	0.35	-	-	-
32.217	Hexadecamethylcyclooctasiloxane	-	-	-	-	-	-	-	0.21	0.39
32.414	1-(3-Methyl-2-butenoxy)-4-(1-propenyl)benzene	-	-	6.23	-	-	-	-	-	-
32.465	Asarone	-	-	-	-	-	8.71	-	-	-
32.971	Santalol, cis,alpha-	-	-	-	-	-	-	-	0.15	-
34.153	Cedren-13-ol, 8-	-	-	-	-	-	-	-	-	0.16
35.571	alpha-Muurolene-14-ol	-	-	-	-	-	-	-	-	1.77
36.778	4,6,6-Trimethyl-2-(3-methylbuta-1,3-dienyl)-3-oxatricyclo[5.1.0.0(2,4)]octane	-	-	-	-	-	-	-	-	2.45
36.928	(E)-1-(2,4,5-Trimethoxyphenyl)but-1-ene	-	-	-	-	-	0.76	-	-	-
37.225	(-)-alpha-Costol	-	-	-	-	-	-	-	-	0.29
37.725	Eremophilone	-	-	-	-	-	-	-	-	0.75
37.887	Methyl isocostate	-	-	-	-	-	-	-	0.24	-
39.787	Octadecamethylcyclononasiloxane	-	-	-	-	-	0.86	-	0.28	0.80
40.28	5-(2-Hydroxy-1-methylethyl)-2-methylcyclohexanone, TBDMS derivative	-	-	0.24	-	-	-	-	-	-
46.01	(3R,6R)-3-Hydroperoxy-3-methyl-6-(prop-1-en-2-yl)cyclohex-1-ene	-	0.15	-	-	-	-	-	-	-
46.817	Eicosamethylcyclodecasiloxane	-	-	-	-	-	-	-	-	1.06
46.87	rel-(+)-(2R,4R,5S)-2,4-Bis(4-methoxyphenyl)-5-methyl-1,3-dioxolane	-	-	0.55	-	-	-	-	-	-
46.904	Octasiloxane, 1,1,3,3,5,5,7,7,9,9,11,11,13,13,15,15-hexadecamethyl-	-	-	-	-	-	1.21	-	-	-
48.789	Cambridge id 5364929	-	-	0.22	-	-	-	-	-	-
52.38	2,4-Bis(4-methoxyphenyl)-3,5-dimethyltetrahydrofuran	-	-	0.22	-	-	-	-	-	-
53.663	Desoximetasone	-	-	0.26	-	-	-	-	-	-
56.043	Triamcinolone	-	-	0.25	-	-	-	-	-	-
59.867	AC1LB8OT	-	-	-	-	-	1.42	-	-	2.15
60.55	Cholest-22-ene-21-ol, 3,5-dehydro-6-methoxy-, pivalate	-	-	-	-	-	0.56	-	-	-
60.776	9,10-Epoxystearic acid, cis-	-	-	-	-	-	0.73	-	-	-
61.203	Decanoic acid, ester with 1,2,3-propanetriol octanoate	-	-	-	-	-	6.15	-	-	0.32
61.389	1,3-Dioctanoyl glycerol	-	-	-	-	-	3.86	-	-	-
61.645	1,2-Dioctanoyl-sn-glycerol	-	-	-	-	-	7.11	-	-	-
	Total	99.14	93.67	99.48	9.68	1.84	98.75	13.46	27.16	96.71

## Data Availability

The original contributions presented in this study are included in the article. Further inquiries can be directed to the corresponding authors.

## References

[1] Shi C., Zhao X.C., Yan H.Y., Meng R.Z., Zhang Y., Li W.L., Liu Z.H., Guo N. (2016). Effect of tea tree oil on *Staphylococcus aureus* growth and enterotoxin production. Food Control.

[2] Gao Z., Zhong W., Chen K., Tang P., Guo J. (2020). Chemical composition and anti-biofilm activity of essential oil from *Citrus medica* L. var. *sarcodactylis* Swingle against *Listeria monocytogenes*. Ind. Crops Prod..

[3] Hua Z., Zhu M.J. (2024). Comprehensive strategies for controlling *Listeria monocytogenes* biofilms on food-contact surfaces. Compr. Rev. Food Sci. Food Saf..

[4] Xiao W., Gao Z., Liu T., Zhong W., Jiang S., He M., Fu F., Li G., Su D., Guo J. (2024). Lemon essential oil nanoemulsions: Potential natural inhibitors against *Escherichia coli*. Food Microbiol..

[5] Agregán R., Munekata P.E.S., Zhang W.G., Zhang J., Pérez-Santaescolástica C., Lorenzo J.M. (2021). High-pressure processing in inactivation of *Salmonella* spp. in food products. Trends Food Sci. Technol..

[6] Wang S.C., Zhao Y.T., Breslawec A.P., Liang T.T., Deng Z.F., Kuperman L.L., Yu Q.N. (2023). Strategy to combat biofilms: A focus on biofilm dispersal enzymes. npj Biofilms Microbiomes.

[7] Guo J.J., Gao Z.P., Xia J.L., Ritenour M.A., Li G.Y., Shan Y. (2018). Comparative analysis of chemical composition, antimicrobial and antioxidant activity of citrus essential oils from the main cultivated varieties in China. LWT-Food Sci. Technol..

[8] Lucia A., Guzmán E. (2021). Emulsions containing essential oils, their components or volatile semiochemicals as promising tools for insect pest and pathogen management. Adv. Colloid Interface Sci..

[9] Khwaza V., Aderibigbe B.A. (2025). Antibacterial Activity of Selected Essential Oil Components and Their Derivatives: A Review. Antibiotic.

[10] Srinivasan R., Jin X.K., Lin X.M., Zhao Z. (2025). Essential Oil Compounds as Antibiotic Alternatives: A Comprehensive Review of Antibacterial, Anti-Quorum Sensing, and Antibiofilm Effects Against Vibrio spp. in Aquaculture. Rev. Aquac..

[11] Xiao W.B., Guo J., Liao Y., Fu Y.J., Li Z., Zhang T.P., Liu T., Xiao Y.B., Shang X.B., Gao Z.P. (2026). The role of geography and regional climate in shaping the chemical profiles and biological potentials of Citrus essential oils. Food Chem..

[12] Xiao W.B., Guo J.J., Fu Y.J., Li Z.X., Zhang T.P., He M.W., Liu T., Xiao Y.B., Shang X.B., Fu F.H. (2025). Long-term study of Citrus changshan-huyou YB Chang essential oil: Chemical transformations and their impact on antibacterial efficacy. Food Chem..

[13] Yin S.L., Xiao Z.Y., Yu Z.J., Zhou C.Y., Jian J.X., Xiao Y.H., Yang H. (2026). Iterative enrichment cultivation and multiomic analysis reveal potential endophytic bacteria affecting the sinomenine synthesis in *Sinomenium acutum*. Microb. Cell Fact..

[14] Xiao W., Xiao Z., Zhang Y., Zhang X., Zeng X., Yu Z., Zeng W., Yin S., Hu R., Chen X. (2026). Multi-omics reveals organ-specific sinomenine biosynthesis and endophyte-mediated modulation in dioecious *Sinomenium acutum*. Ind. Crops Prod..

[15] Xiang F., Bai J.H., Tan X.B., Chen T., Yang W., He F. (2018). Antimicrobial activities and mechanism of the essential oil from *Artemisia argyi* Levl. et Van. var. argyi cv. Qiai. Ind. Crops Prod..

[16] Duan H., Wang W., Shi Y., Wang L., Khan G.J., Luo M., Zhou J., Yang J., He C., Li F. (2025). Anti-colorectal cancer actions of *Glycyrrhiza uralensis* Fisch. and its underlying mechanism via HPLC integration and network pharmacological approaches. Phytomedicine.

[17] Liu Y.C., Xu H.S., Chen Z.Y., Xie Z.Y., Wen H., Chang X., Li G.Y. (2025). Development of Lily Starch Films Reinforced with Chitosan-Honeysuckle Essential Oil Hybrid Particles and Cellulose Nanofibers for Enhanced Properties. Foods.

[18] Cattò C., de Vincenti L., Borgonovo G., Bassoli A., Marai S., Villa F., Cappitelli F., Saracchi M. (2019). Sub-lethal concentrations of *Perilla frutescens* essential oils affect phytopathogenic fungal biofilms. J. Environ. Manag..

[19] Hu Z.Y., Yuan K., Zhou Q., Lu C., Du L.H., Liu F. (2021). Mechanism of antifungal activity of *Perilla frutescens* essential oil against *Aspergillus flavus* by transcriptomic analysis. Food Control.

[20] Li W., Ding J., Chen S., Chen J., Wang C., Li J., Shi H., Yin X., Wang J., Liu J. (2024). Alleviation of colitis by honeysuckle MIR2911 via direct regulation of gut microbiota. J. Control. Release.

[21] Liu S., Dong H.J., Lu H., Li Y.F., Zhang G.H., Mi Y.Z., Wang X. (2025). Dynamic changes in composition during processing of *Lonicerae japonicae* flos black tea. Food Chem..

[22] Xu P., Zhang Z.T., Peng X.Y., Yang J.L., Li X.Q., Yuan T.J., Jia X.H., Liu Y.Y., Abdullaev O., Jenis J. (2022). Study on vacuum drying kinetics and processing of the *Lonicera japonica* Thunb. aqueous extracts. LWT-Food Sci. Technol..

[23] Gao Z., Jiang S., Zhong W., Liu T., Guo J. (2023). Linalool controls the viability of *Escherichia coli* by regulating the synthesis and modification of lipopolysaccharide, the assembly of ribosome, and the expression of substrate transporting proteins. Food Res. Int..

[24] He M., Zhong W., Dai R., Long S., Zhou Y., Zhang T., Zhou B., Tang T., Yang L., Jiang S. (2025). Linalool exhibit antimicrobial ability against *Elizabethkingia miricola* by disrupting cellular and metabolic functions. Curr. Res. Microb. Sci..

[25] Noshirvani N. (2024). Essential Oils as Natural Food Preservatives: Special Emphasis on Antimicrobial and Antioxidant Activities. J. Food Qual..

[26] Rahman A., Kang S.C. (2009). In vitro control of food-borne and food spoilage bacteria by essential oil and ethanol extracts of *Lonicera japonica* Thunb. Food Chem..

[27] Falleh H. (2025). Demystifying the power of essential oils: A review of their antibacterial properties and potential as natural food preservatives. EXCLI J..

[28] Li R.Z., Guan X.X., Wang X.R., Bao W.-Q., Lian L.-R., Choi S.W., Zhang F.Y., Yan P.-Y., Leung E.L.H., Pan H.-D. (2023). Sinomenine hydrochloride bidirectionally inhibits progression of tumor and autoimmune diseases by regulating AMPK pathway. Phytomedicine.

[29] Zhao Q., Chen X.Y., Martin C. (2016). Scutellaria baicalensis, the golden herb from the garden of Chinese medicinal plants. Sci. Bull..

[30] Huang X.H., Chen W.J., Yang S.Y., Wang B., Hu X.Y., Dong Z.L., Zhang J.M. (2025). Honeysuckle Extracts (*Lonicera japonica* Thunb.): Understanding Insights into the Antioxidant Effect on Preserving Qualities of Rabbit Meat During Refrigerated Storage. Foods.

[31] Latorre R., Valerii M.C., Benati M., Lewis R.E., Spigarelli R., Bernacchi A., Lippi G., Spisni E., Gaibani P. (2025). Lights and Shadows of Essential Oil-Derived Compounds: Antimicrobial and Anti-Inflammatory Properties of Eugenol, Thymol, Cinnamaldehyde, and Carvacrol. Curr. Issues Mol. Biol..

[32] Pang D.R., Huang Z.X., Li Q., Wang E., Liao S., Li E., Zou Y.X., Wang W.F. (2021). Antibacterial Mechanism of Cinnamaldehyde: Modulation of Biosynthesis of Phosphatidylethanolamine and Phosphatidylglycerol in *Staphylococcus aureus* and *Escherichia coli*. J. Agric. Food Chem..

[33] Tang T., Zhong W., Tang P., Dai R., Guo J., Gao Z. (2025). Linalool combats Saprolegnia parasitica infections through direct killing of microbes and modulation of host immune system. eLife.

[34] Tang T., Zhong W.M., Yang L.L., He M.W., Jiang S.F., Yin D., Guo J.J., Gao Z.P. (2024). In vitro and in vivo anti-oomycetes activities and mechanisms of linalool against *Saprolegnia ferax*. Aquaculture.

[35] Gao Z., Zhong W., Liu T., Zhao T., Guo J. (2021). Global Proteomic Analysis of *Listeria monocytogenes*’ Response to Linalool. Foods.

[36] Iskandar K., Ahmed N., Paudyal N., Alvarez M.-J.R., Balasubramani S.P., Saadeh D., Baig S.U., Sami H., Halat D.H., Pavlović N. (2025). Essential Oils as Antimicrobial Agents Against WHO Priority Bacterial Pathogens: A Strategic Review of In Vitro Clinical Efficacy, Innovations and Research Gaps. Antibiotics.

[37] Grooters K.E., Ku J.C., Richter D.M., Krinock M.J., Minor A., Li P., Kim A., Sawyer R., Li Y. (2024). Strategies for combating antibiotic resistance in bacterial biofilms. Front. Cell. Infect. Microbiol..

[38] Reem C.S.A., Chowdhury M.A.H., Ashrafudoulla M., Rahman M.A., Yoon H.J., Ha S.D. (2025). Advancing biofilm prevention in the food industry to combat food degradation: A bibliometric analysis on the perspective of food safety. Food Control.

[39] Guo J., Hu X., Gao Z., Li G., Fu F., Shang X., Liang Z., Shan Y. (2021). Global transcriptomic response of *Listeria monocytogenes* exposed to Fingered Citron (*Citrus medica* L. var. *sarcodactylis* Swingle) essential oil. Food Res. Int..

[40] He J., Fan Z., Jiang Q., Yang X., Sun J., Pang Y., Fang X., Zhang D., Li Y., Liu Y. (2025). Sustainable natural bio-antimicrobial: Composition; bacteriostatic activity, and antibacterial strategy of *Cinnamomum camphora* essential oil. Ind. Crops Prod..

[41] Pierozan M.B., de Oliveira J.G., Cappato L.P., Costa A.C., Egea M.B. (2024). Essential Oils Against Spoilage in Fish and Seafood: Impact on Product Quality and Future Challenges. Foods.

